# CellPrior-net: Prior-guided nuclei detection and classification for H&E whole-slide images

**DOI:** 10.1016/j.jpi.2026.100716

**Published:** 2026-08-31

**Authors:** Falah Jabar, Pasquale Lombardi, Aria Torkpour, Masoud Tafavvoghi, Per Niklas Benzler Waaler, Sigve Andersen, Erna-Elise Paulsen, Mette Pøhl, Lill-Tove Rasmussen Busund, Tom Donnem, Elin Richardsen, David J. Pinato, Mehrdad Rakaee

**Affiliations:** a Dep. of Clinical Pathology, University Hospital of North Norway, Tromsø, Norway; b Dep. of Surgery and Cancer, Imperial College London, London, United Kingdom; c Department of Medical Biology, UiT The Arctic University of Norway, Tromsø, Norway; d Dep. of Clinical Medicine, UiT The Arctic University of Norway, Tromsø, Norway; e Dep. of Pulmonology, University Hospital of North Norway, Tromsø, Norway; f Dep. of Clinical Medicine, University of Copenhagen, Copenhagen, Denmark; g Dep. of Cancer Genetics, Oslo University Hospital, Oslo, Norway

**Keywords:** Computational pathology, Computer vision, Digital pathology, Deep learning, Nuclei detection and classification, H&E whole-slide images

## Abstract

Accurate nuclei detection and classification in hematoxylin and eosin (H&E) whole-slide images (WSIs) is a key task in computational pathology, particularly for quantitative analysis of the tumor microenvironment. However, this task remains highly challenging due to variations in nuclei morphology, staining procedures, scanners, organs, magnifications, and WSI artifacts. In addition, many existing pipelines rely on computationally demanding architectures and post-processing procedures, making gigapixel WSI analysis time-consuming. In this work, CellPrior-Net (CP-Net) is proposed, an efficient nuclei detection and classification pipeline that utilizes a lightweight convolutional neural network architecture and hematoxylin (H) channel as prior information to enhance nuclei-aware feature learning. Extensive benchmarking was conducted against state-of-the-art pipelines on eight public and private datasets (total:∼10.4 M nuclei) obtained from different organs, scanners, magnifications, and clinical centers. Experimental results demonstrate that CP-Net achieves comparable performance while significantly reducing inference time. Furthermore, CellQuant-Net was introduced—an end-to-end nuclei quantification pipeline—that integrates a quality assessment model to exclude regions with artifacts, followed by CP-Net cell detection and classification. The pipeline is publicly available on GitHub, and provides a potentially efficient and scalable framework for downstream computational pathology applications.

## Highlights

• Development of a lightweight nuclei detection and classification pipeline that utilizes both H&E images and the H channel as prior information to classify nuclei into three categories: immune, tumor, and other cells.
• Extensive benchmarking experiments against state-of-the-art pipelines using eight public and private datasets collected from different organs, scanners, magnifications, and both single- and multi-organ WSIs.
• Development of CellQuant-Net that integrate an QA pipeline with CP-Net to first remove artifacts from WSIs before nuclei detection and classification, followed by a nuclei connectivity analysis to model nuclei spatial relationships. Available on GitHub.

## Graphical abstract

**Figure:**
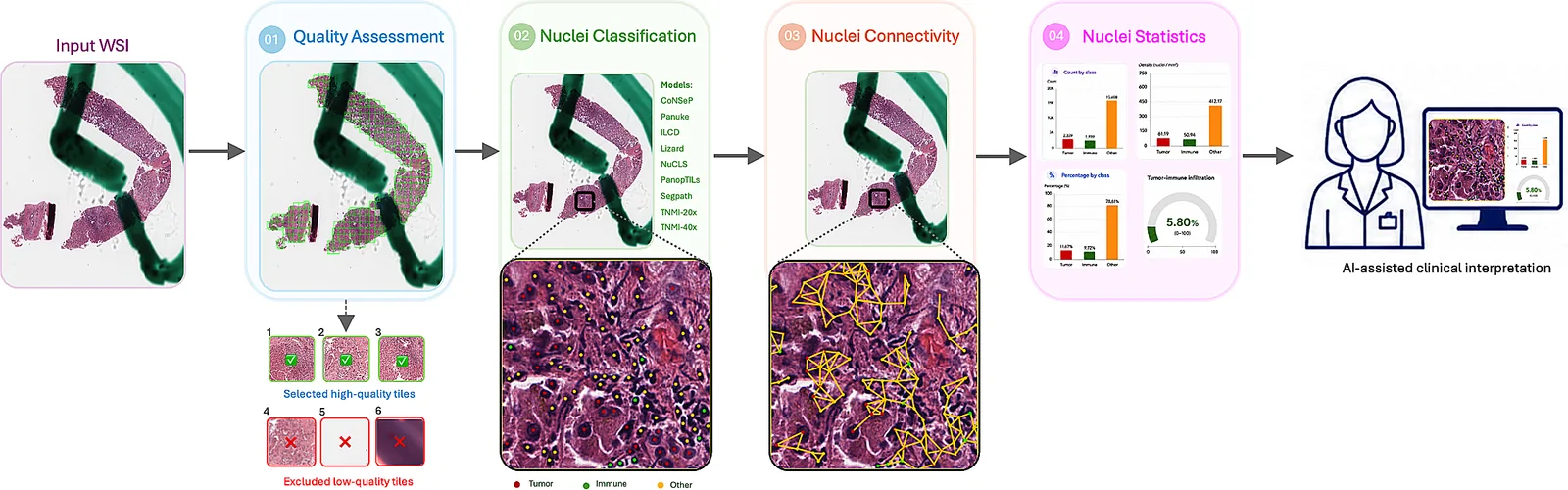

## 1 Introduction

Whole-slide imaging (WSI) enables the digitization of histopathology slides into gigapixel WSIs, providing detailed histological information. Because WSIs are extremely large and cannot be directly processed by deep learning (DL) models, they are divided into smaller image tiles (Fig. 1). These tiles are subsequently used as input for various DL tasks, such as quantification, histological subtyping, genomic alterations, and treatment outcome prediction.^1^, ^2^, ^3^

**Fig. 1:**
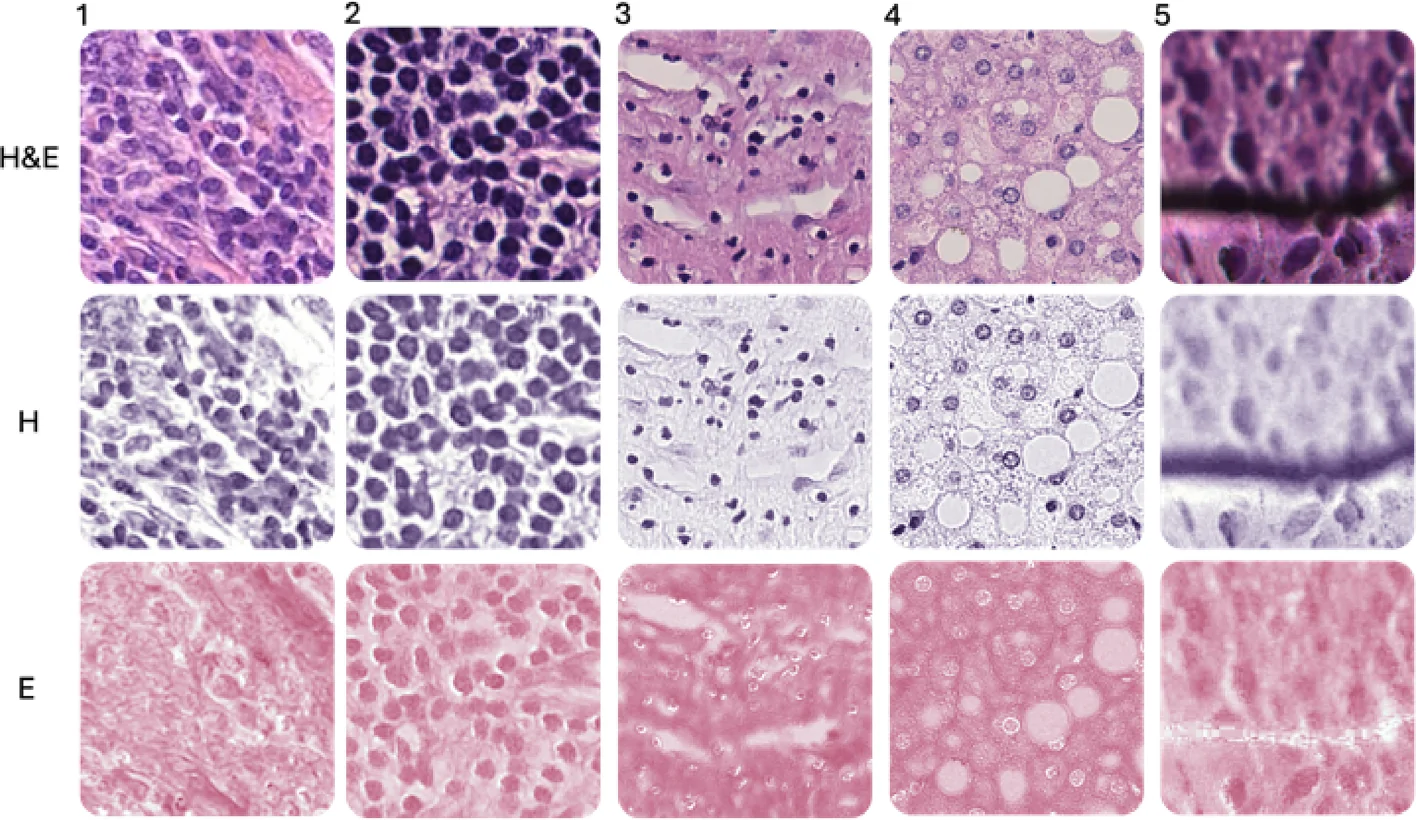
Examples showing the variability in nuclei appearance in H&E WSIs. Columns 1–5 present different tissue and nuclei patterns extracted from multiple WSIs. The first row shows the original H&E image tile, whereas the second and third rows show the separated hematoxylin (H) and eosin (E).

Several pathology foundation models (FMs) have been developed for computational pathology applications.^4^, ^5^, ^6^, ^7^, ^8^, ^9^, ^10^, ^11^, ^12^, ^13^, ^14^, ^15^, ^16^ In particular, nuclei detection and classification have become a primary task, as they enable quantitative analysis of the tumor microenvironment and immune infiltration. Thus, several DL pipelines for nuclei detection and classification have been proposed.^17^, ^18^, ^19^, ^20^, ^21^, ^22^, ^23^, ^24^, ^25^

However, up to date, accurate nuclei detection and classification remain highly challenging due to several factors (Fig. 1):

• *Morphology:* Differences in nuclei shape, size, texture, and appearance across tissues and cancer grades.
• *Organs:* Different organs have diverse tissue architectures, cell distributions, and appearances.
• *Staining:* Differences in staining procedures between labs introduce color and contrast inconsistencies.
• *Slicing:* During tissue slicing, some nuclei may appear partially visible or hidden beneath the tissue slice.
• *Scanner:* Different scanners produce variations in resolution, color distribution, sharpness, and illumination.
• *Overlapping*: Separating touching nuclei is challenging, particularly in regions with unclear nuclei boundaries.
• *Artifacts:* Artifacts (e.g., folds, blur, pen markings) may partially or completely obscure nuclei regions.
• *Magnification:* Nuclei may appear with different sizes and appearances at 20× versus 40× magnification.
• *Gigapixel WSI***:** Processing gigapixel WSIs is computationally expensive, especially for nuclei quantification.
• *Dataset annotation:* Nuclei annotation is highly challenging and time-consuming due to manual labeling.

Some of these challenges introduce domain shifts that cause the model to fail frequently on unseen data.^19^ Also, most of the existing pipelines require considerable computational resources to process a single WSI due to complex model architecture and computationally expensive post-processing procedures. For example, processing a single high-resolution WSI may take several hours, or even days in some cases.^25^ Moreover, it remains unclear how well existing nuclei detection and classification pipelines generalize across different datasets, organs, scanners, magnifications, and clinical centers. Many pipelines rely on post-processing to separate touching nuclei using predicted nuclei edge masks and may often fail for accurate classification at 20× magnification, as nuclei appear denser and more clustered (Fig. 3). Furthermore, none of these pipelines consider quality assessment (QA) to exclude regions with artifacts before or during nuclei detection and classification. As shown in works,^26^, ^27^, ^28^ artifacts can significantly reduce nuclei detection and classification accuracy.

Together, these limitations highlight the need for WSI-scale nuclei analysis pipelines that integrate QA, accurate nuclei detection and classification, and computational efficiency. This is particularly important because nuclei analysis requires dense instance-level processing of gigapixel WSIs, whereas most existing pipelines operate directly on full hematoxylin and eosin (H&E) images despite nuclei being predominantly represented in the hematoxylin component. Separating H&E components can be beneficial as the H channel enhances nuclei regions while reducing background noises (Fig. 1). In this context, the main objective of this article is to develop a lightweight nuclei detection and classification pipeline, CellPrior-Net (CP-Net), which utilizes the hematoxylin (H) channel as prior information to guide the network toward nuclei-relevant features, whereas using an efficient post-processing procedure that generalizes across different magnifications.

The main contributions of this work are:

• Development of the ILCD dataset, collected from 40 liver cancer WSIs at Imperial College London, annotated manually by experts pathologists. The dataset contains three cell categories: immune, tumor, and other cells (detailed in Supplementary Material).
• Development of a lightweight nuclei detection and classification pipeline that utilizes both H&E images and the H channel as prior information to classify nuclei into three categories: immune, tumor, and other cells (Fig. 2).

**Fig. 2:**
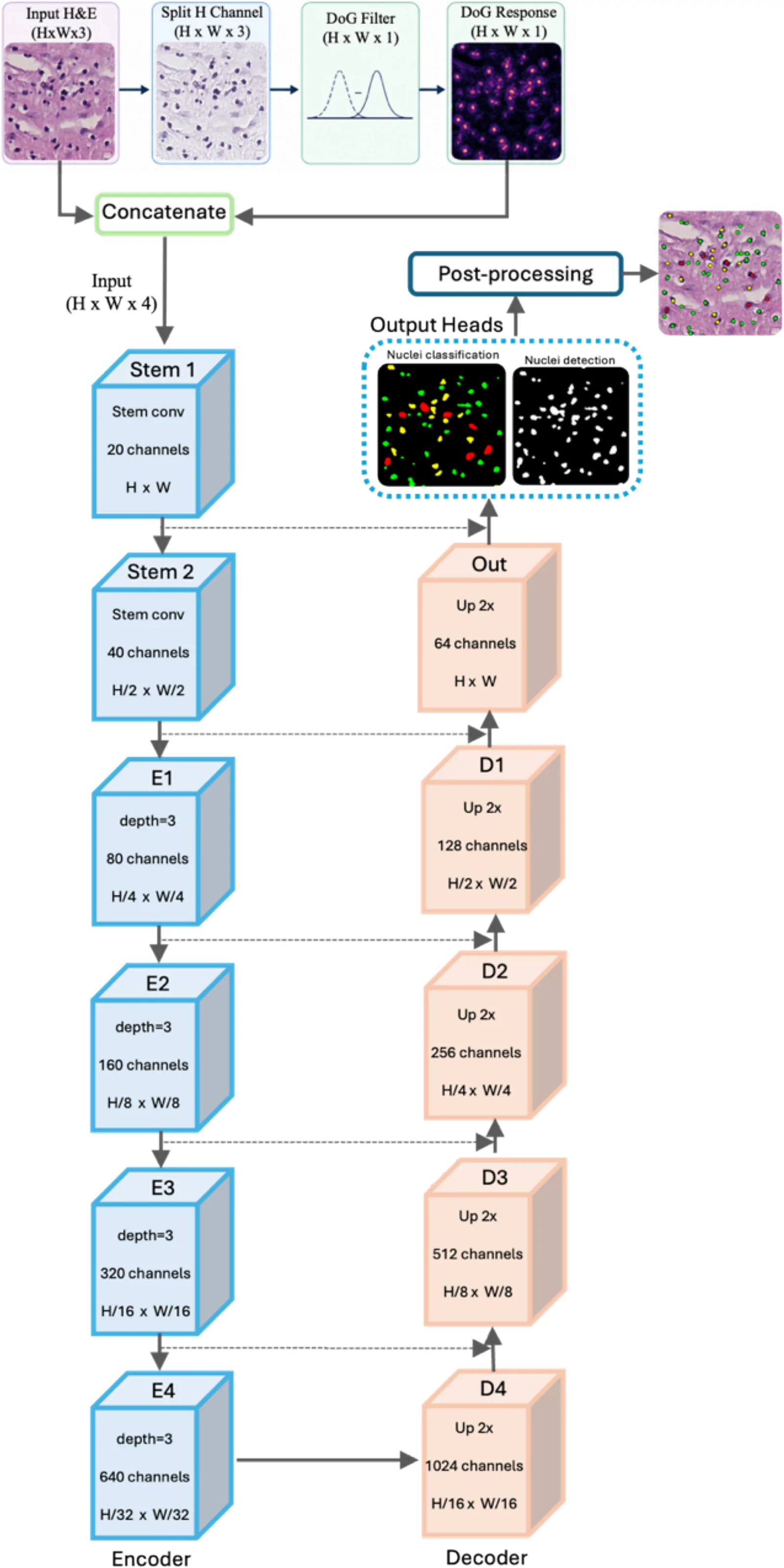
Overview of the CP-Net architecture.
• Extensive benchmarking experiments against state-of-the-art pipelines using eight public and private datasets collected from different organs, scanners, magnifications, and both single- and multi-organ WSIs (detailed in Supplementary Material).
• Integration of a QA pipeline with CP-Net to first remove artifacts from WSIs before nuclei detection and classification, followed by a nuclei connectivity analysis to model nuclei spatial relationships (Fig. 5).

The rest of this article is organized as follows: Section II reviews related work, Section III describes the datasets, Section IV presents the proposed CP-Net methodology, Section V reports the performance evaluation results, Section VI introduces CellQuant-Net, and Section VII concludes the article and discusses future directions.

## 2 Related work

Nuclei detection and classification pipelines have evolved from convolutional neural network (CNN)-based to vision transformer (ViT)-based and FM-based approaches. In Graham et al.,^22^ HoVer-Net was introduced as one of the earliest CNN-based approaches, utilizing three decoder branches with a shared encoder to perform nuclei separation, detection, and classification in H&E WSIs. The post-processing step of the three output branches generates the final detected and classified nuclei. TSFD-Net, a tissue-specific feature distillation pipeline for nuclei detection and classification, was proposed in Ilyas et al.^25^ TSFD-Net incorporated a tissue-specific feature distillation backbone together with a bidirectional feature pyramid network and interlinked decoders for hierarchical feature fusion and joint optimization. The framework predicts nuclei classification and boundary masks, followed by post-processing to generate the final output. Cerberus^23^ is a multi-task learning framework with a shared encoder and task-specific decoders, enabling the prediction of nuclei, glands, lumen, and tissue regions in H&E WSIs. The architecture consists of four dedicated decoder branches, where each decoder is optimized for a specific task while sharing a common encoder representation for efficient feature learning and joint optimization. CellViT^18^ introduced a ViT-based encoder architecture in which the backbone was integrated with three decoder branches through skip connections to jointly learn nuclei separation, detection, and classification. The outputs from these branches were fused through a post-processing stage to obtain the final output. Inspired by CellViT, CellViT++^19^ utilized an FM-based encoder to generate nuclei detection masks and deep nuclei feature embeddings, which were subsequently used by a lightweight classifier for nuclei classification. Similar to CellViT, NuLite^29^ employed a lightweight U-Net-based encoder–decoder to reduce computational cost and inference time. The architecture utilized three decoder branches dedicated to nuclei detection, classification, and separation. LKCell^20^ also utilized a U-Net-based encoder–decoder architecture with large-kernel convolutions and three segmentation heads dedicated to nuclei detection, classification, and separation. PointNu-Net^24^ utilizes a high-resolution network backbone followed by three task-specific branches: a heatmap branch, a kernel branch, and a feature branch. These branches were integrated during post-processing to generate the final detected and classified nuclei. CellSAM^17^ is a FM-based pipeline built on top of the Segment Anything Model.^30^ It combines an automatic cell detector, CellFinder, with SAM to detect multiple cell types, organisms, and microscopy modalities using a single unified model. However, the framework focuses on cell detection, without performing cell classification. CellDETR^21^ uses a hierarchical swin transformer backbone to extract multi-scale feature representations. The extracted features are flattened and enriched with positional and level embeddings before being processed by a transformer encoder using multi-scale attention layers, detecting and classifying nuclei.

More details about the state-of-the-art pipelines are provided in the supplementary material (Table S2). Despite recent advances, several limitations remain in existing nuclei detection and classification pipelines. Many pipelines are trained and evaluated using intra-dataset settings, using train/test splits or cross-validation. Consequently, their cross-dataset generalizability remains unclear. In addition, benchmarking different pipelines remains difficult due to variations in datasets and predicted nuclei classes.

## 3 Data material

In this work, several H&E-stained datasets were used, namely CoNSeP,^22^ Lizard,^31^ NuCLS,^32^ PanopTILs,^33^ SegPath,^34^ PanNuke,^35^ and TNMI.^3^ In addition, the ILCD dataset is proposed as part of this work. A detailed description of each dataset is provided in the Supplementary Material. These datasets differ in terms of organ, scanner, magnifications, annotation procedure, cell categories, and tile sizes. The SegPath dataset does not provide cell-level annotations; therefore, additional preprocessing procedures were applied to generate cell-level annotations and standardize all image tiles to a size of 256 × 256 pixels across datasets. Furthermore, all datasets were unified into three common cell categories by merging the original labels into immune cells, tumor cells, and other cells. This allows a fair and consistent evaluation of the different pipelines across multiple datasets.

## 4 Method

The overview of the proposed pipeline is presented in Fig. 2. Given an input H&E image tile, the hematoxylin (H) component is first separated and processed with a multi-scale Difference of Gaussians (DoG) filter to identify nuclei regions and generate a DoG mask. The DoG mask is then concatenated with the H&E image to obtain a 4-channel input, which is fed into the model. The model produces both a binary nuclei segmentation mask and a nuclei type classification mask. Finally, a post-processing step is applied to refine the boundaries of nuclei and generate the final predictions, where nuclei are detected and classified into tumor, immune, and other types. The main step of the pipeline is described below.

### 4.1 H component separation

Given a H&E image $I\in R^{H\times W\times 3}$, the image is first converted into optical density space^36^:

(1) $$O=-\log\left(\frac{I+1}{I_{o}}\right),$$

where $I_{o}=240$ is the transmitted light intensity, and the constant 1 avoids numerical instability from zero-pixel values. Pixels with low optical density values are removed using a threshold β=0.15:

(2) $$\hat{O}=O\;\left[O\geq \beta \right].$$

The covariance matrix of the optical density matrix is computed, and then eigenvalue decomposition:

(3) $$\Sigma =\mathrm{Cov}\left({\hat{O}}^{T}\right),$$

(4) Σv=λ.v,

where Σ denotes the covariance matrix, v is an eigenvector, and λ is an eigenvalue. The $\hat{O}$ values are then projected onto this two-dimensional (2D) stain plane, and then the angular distribution of the projected pixels is computed:

(5) $$\Theta =\hat{O}.v,$$

(6) $$\varphi =\arctan2\left(\Theta \right).$$

The minimum and maximum stain vectors are estimated using the 1 and 99 percentiles of the angular distribution.

(7) $$\varphi_{\min}=\text{percentile}\left(\varphi ,1\right),$$

(8) $$\varphi_{\max}=\text{percentile}\left(\varphi ,99\right).$$

The two stain vectors are computed:

(9) $$v_{\min}=v\;\left[\cos\left(\varphi_{\min}\right),\sin\left(\varphi_{\min}\right)\right],$$

(10) $$v_{\max}=v\;\left[\cos\left(\varphi_{\max}\right),\sin\left(\varphi_{\max}\right)\right].$$

The final stain matrix $M_{\mathrm{HE}}$ is computed as:

(11) $$M_{\mathrm{HE}}=\left[v_{\min},v_{\max}\right].$$

The optical density matrix O is then decomposed into stain concentrations using least-squares estimation:

(12) $$C=\underset{C}{\text{argmin}}{\left\|\;O^{T}-M_{\mathrm{HE}}.C\right\|}^{2}.$$

The first row of C, denoted as $C_{H}$, corresponds to the hematoxylin concentration. Then, the hematoxylin concentration is normalized using the 99th percentile:

(13) $$C_{H,\max}=\text{percentile}\left(C_{H},99\right),$$

(14) $$C_{H,\mathrm{norm}}=\frac{C_{H}}{C_{H,\max}/C_{H,\mathrm{ref}}},$$

where $C_{H,\mathrm{ref}}=1.9705$ is the reference maximum hematoxylin concentration. Finally, the hematoxylin image is reconstructed using the hematoxylin reference stain vector:

(15) $$H=I_{o}\exp\left(-H_{\mathrm{ref}}.C_{H,\mathrm{norm}}\right),$$

where $H_{\mathrm{ref}}$ is the hematoxylin column of the reference stain matrix ${\left[0.56260.72010.4062\right]}^{T}$. The reconstructed H image (Fig. 2) is then reshaped back to the original image size (H×W×3).

### 4.2 Difference of Gaussian (DoG)

Given the extracted H component, the next step is to convert the H image to grayscale $H_{g}\in R^{H\times W}$. Gaussian filtering is then defined as:

(16) $$L_{\sigma }\left(H\right)=G_{\sigma }*H_{g},$$

(17) $$G_{\sigma }\left(n,m\right)=\frac{1}{2\pi \;\sigma^{2}}\exp\left(-\frac{n^{2}-m^{2}}{2\;\sigma^{2}}\right),$$

where * denotes 2D convolution, σ controls the smoothing scale, and $\left(n,m\right)$ denotes the spatial coordinates on $H_{g}$. For each Gaussian scale σ, the DoG response is computed by subtracting two Gaussian-smoothed versions of the $H_{g}$ image:

(18) $$D_{\sigma }\left(H\right)=L_{\sigma }\left(H\right)-L_{\mathrm{k\sigma}}\left(H\right),$$

where $k=\sqrt{2}\;$is the scale ratio between the two Gaussian kernels. DoG response is computed over a predefined set of Gaussian scales S. The final DoG mask is obtained by selecting the maximum response across all scales:

(19) $$D\left(H\right)=\max_{\sigma \in S}D_{\sigma }\left(H\right),\mathrm{where}\;S=\left\{\begin{array}{c} \left\{2.53.5,4.5\right\}\;\text{for}\;40x\\ \left\{1.62.4,3.2\right\}\;\text{for}\;20x \end{array}\right),$$

where the scale set S is defined according to the image magnification and obtained through several experiments for both 20× and 40× magnifications.

### 4.3 Model architecture

As shown in Fig. 2, the four-channel input is fed to a UniRepLKNet-N encoder,^37^ which extracts hierarchical feature representations. The encoder consists of four stages. Each stage progressively downsamples the spatial resolution while increasing the feature dimensions through large-kernel depth-wise convolutions. The decoder follows a U-Net-like architecture with skip connections between the encoder and decoder stages. High-level encoder features are progressively upsampled and fused with lower-level encoder features using concatenation blocks. Finally, two independent segmentation heads are attached to the decoder output. The first head predicts the binary nuclei segmentation mask, whereas the second head predicts the nuclei type classification mask. Following Hörst et al.,^18^ the overall objective function is defined as:

(20) $$L_{\mathrm{total}}=L_{\mathrm{type}}+L_{\det},$$

where $L_{\mathrm{type}}$ and $L_{\det}$ denote the nuclei type classification and nuclei detection losses, respectively. The nuclei type classification and nuclei detection losses are defined as:

(21) $$L_{\mathrm{type}}=\lambda_{\mathrm{CE}}^{\mathrm{type}}L_{\mathrm{CE}}^{\mathrm{type}}+\lambda_{\mathrm{Dice}}^{\mathrm{type}}L_{\mathrm{Dice}}^{\mathrm{type}}+\lambda_{\mathrm{FT}}^{\mathrm{type}}L_{\mathrm{FT}}^{\mathrm{type}}$$

(22) $$L_{\det}=\lambda_{\mathrm{Dice}}^{\det}L_{\mathrm{Dice}}^{\det}+\lambda_{\mathrm{FT}}^{\det}L_{\mathrm{FT}}^{\det},$$

where $L_{\mathrm{CE}}^{\mathrm{type}}$, $L_{\mathrm{Dice}}^{\mathrm{type}}$, and $L_{\mathrm{FT}}^{\mathrm{type}}$ denote the cross-entropy, Dice, and Focal Tversky losses for nuclei type classification, respectively, whereas $L_{\mathrm{Dice}}^{\det}$ and $L_{\mathrm{FT}}^{\det}$ denote the Dice and Focal Tversky losses for nuclei detection. The weighting parameters λ control the contribution of each loss component during training and were set similarly to Hörst et al.^18^ The Adam optimizer was employed to optimize the proposed loss function and train the network parameters. A hyperparameter search was conducted by evaluating different training configurations, including the learning rate, batch size, and number of epochs, aiming for the minimum loss, and achieving the best overall performance. The best-performing configuration consisted of 150 training epochs, a batch size of 24, and an initial learning rate of $1\times 10^{-3}.$ TensorBoard visualization tool was used to monitor the training losses, learning progress, and convergence behavior throughout the training process. To improve model robustness and generalization, several data augmentation techniques were applied during training, including random horizontal and vertical flipping, random 90° rotations, color jittering, and Gaussian blurring. Training was performed on an Ubuntu 20.04 operating system, a 40-core CPU, and a 512 GB of RAM workstation equipped with an NVIDIA A100 GPU with 80 GB of memory running CUDA 12.2. To maintain consistency with existing pipelines, which are commonly trained on the multi-organ PanNuke dataset,^35^ CP-Net was also trained on the PanNuke dataset (40× magnification, 0.25 μm/px). However, unlike most existing methods that employ 3-fold cross-validation and report the best-performing model, CP-Net was trained using the entire PanNuke dataset and subsequently evaluated on independent external datasets for benchmarking, as detailed in Section V.

### 4.4 Post-processing

Consider the predicted nuclei mask B for a given H&E image tile I, where 1: nuclei pixels and 0: background pixels. The following procedure was developed to separate touching nuclei and generate the final nuclei segmentation mask:

1) *Morphological erosion*: Apply the morphological erosion to separate weakly connected nuclei and reduce boundary noise:

(23) $$B_{e}=B⊖\tilde{S},$$

where $B_{e}$ is the eroded binary mask, ⊖ denotes morphological erosion, $\tilde{S}$ and is a structuring element defined by the erosion radius $e_{r}$.

2) *Distance mask:* Compute the distance mask to identify the central regions of nuclei:
  (24) $$D_{m}=E\left(B_{e}\right),$$

where $E\left(.\right)$ denotes the Euclidean distance transform function applied to the eroded binary mask $B_{e}$ with a mask size $m_{s}$. Pixels near nuclear centers have larger values than pixels near nuclear boundaries or background regions (Fig. 3).

3) *Local markers:* Extract local markers from the distance mask to initialize watershed segmentation:
  (25) $$M=\hat{L}\left(D_{m}\right),$$

where M represents the set of marker locations and $\hat{L}\left(.\right)$ denotes the peak-local-maxima function, where each detected maximum likely corresponds to the center of a nucleus. The parameter $d_{m}$ controls the minimum distance between neighboring peaks and $t_{\mathrm{abs}}$ filters weak peaks.

4) *Watershed segmentation:* The watershed algorithm was applied to the distance mask to generate the segmentation mask with separated nuclei:
  (26) $$I_{w}=W\left(-D_{m},M,B_{e}\right),$$

where $W\left(.\right)$ denotes the watershed function, and $I_{w}$ represents the final segmentation mask in which each nucleus is assigned a unique label (Fig. 3).

5) *Small object removal:* Small segmented objects with an area smaller than the predefined threshold $a_{\min}$ were removed, as they likely correspond to background noise.
6) *Final segmentation mask*: The final segmentation mask is generated by combining the segmentation mask $I_{w}$ with the predicted nuclei type mask, where each segmented nucleus is assigned to its corresponding class (Fig. 2).

**Fig. 3:**
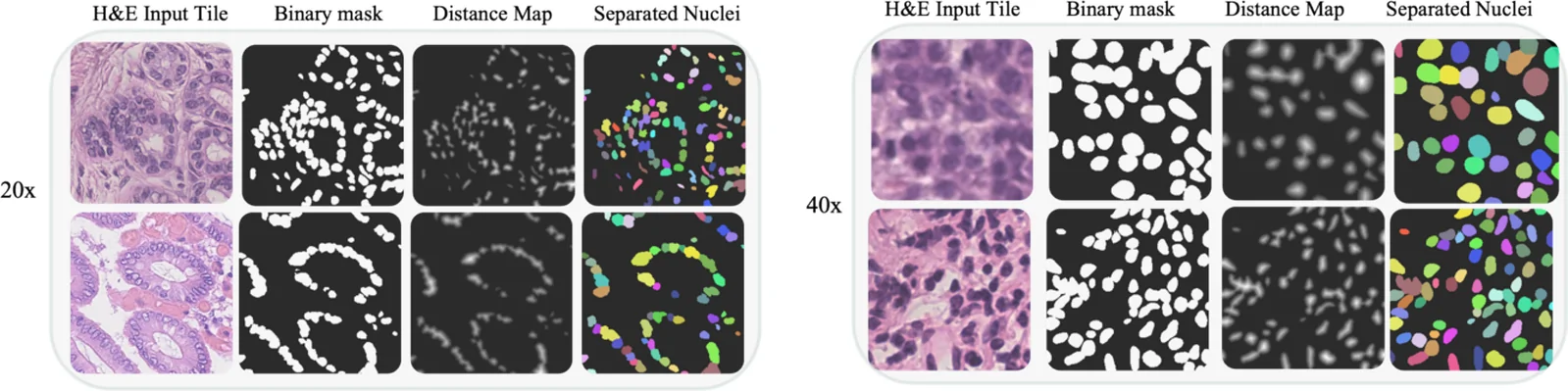
Examples of the nuclei separation procedure (Steps 1–5) for 20× and 40× magnifications. From left to right: input H&E image tile, binary mask, distance mask, and final separated nuclei, where each nucleus is represented by a different color.

This procedure has several hyperparameters that must be carefully chosen. Therefore, a parameter optimization procedure was adopted to determine the optimal set of hyperparameters for each magnification level 20× and 40×. First, 100 binary segmentation masks were carefully selected for each 20× and 40× magnification (a total of 200 masks). The selected masks included diverse nucleus types, densities, and touching-nuclei scenarios from different organs and datasets. Each segmentation mask was manually inspected, and the nuclei were visually counted to obtain the ground-truth number of nuclei, including both touching and non-touching cells. The procedure steps 1–5 were then applied using different hyperparameter combinations, and the following optimization loss function was computed:

(27) $$L=\frac{1}{N}\sum_{i=1}^{N}|C_{i}^{\mathrm{gt}}-C_{i}^{\mathrm{pr}}|,$$

where $C_{i}^{\mathrm{gt}}$ is ground-truth nuclei count, $C_{i}^{\mathrm{pr}}$ represents predicted number of nuclei after step 5, N is the total number of evaluated masks. The optimized hyperparameters for the 20× setting were $e_{r}=1$, $m_{s}=5\times 5$, $d_{m}=4$, $t_{\mathrm{abs}}=2$, and $a_{\min}=10$, and for the 40× setting, $e_{r}=2$, $m_{s}=5\times 5$, $d_{m}=8$, $t_{\mathrm{abs}}=2$, and $a_{\min}=20$. For efficiency, the post-processing procedure was implemented using GPU-based morphological operations and connected component analysis with PyTorch/CuPy, together with multi-threaded watershed segmentation. Fig. 3 presents representative nuclei separation results at both 20× and 40× magnifications, demonstrating that the proposed post-processing procedure effectively separates touching nuclei regardless of the magnification on which the model was trained.

## 5 Performance evaluation

The performance of the CP-Net was evaluated and compared with state-of-the-art methods, including CellViT, CellViT++, Cerberus, HoVer-Net, NuLite, PointNu-Net, CellDetr, CellSAM, LKCell, and TSFD (Table S2 in the Supplementary Materials). The evaluation was conducted on eight datasets, including CoNSeP, ILCD, Lizard, NuCLS, PanopTILs, Segpath, TNMI-20×, and TNMI-40× (Table S1 in the Supplementary Materials). Because different pipelines may predict different nuclei categories, the output classes of all pipelines were merged into three classes: immune cells, tumor cells, and other cells, to allow a consistent comparison. The performance metrics detection quality (DQ), segmentation quality (SQ), and panoptic quality (PQ) were used for evaluation.^18^, ^19^, ^22^ DQ measures the accuracy of nuclei detection, SQ measures the quality of the predicted nuclei segmentation, and PQ is a unified metric that jointly evaluates nuclei detection and segmentation performance, reflecting how well the model detects and classifies nuclei. These metrics were computed separately for each nuclei class and then averaged across all classes.

Fig. 4 presents a comprehensive benchmarking of state-of-the-art pipelines across multiple datasets (a), scanner types (b), nuclei classes (c), magnifications (d), performance vs inference time (e) averaged across all datasets. A qualitative comparison of each dataset and pipeline is provided in Table S4 of the Supplementary Materials. The following conclusion can be drawn:

• *Across datasets*: The DQ, SQ, and PQ metrics for each pipeline and dataset are presented in Fig. 4a: colon (CoNSeP and Lizard), liver (ILCD), breast (NuCLS and PanopTILs), lung (TNMI), and the multi-organ (SegPath). The average performance across datasets ranged from 0.07–0.46 for DQ, 0.22–0.62 for SQ, and 0.05–0.34 for PQ, highlighting the large variability in metric values and the limited generalizability of current pipelines across different datasets. Pipelines trained on one dataset often struggle to generalize to unseen datasets because datasets differ in organ, staining, scanner, and tissue.^17^, ^21^, ^22^ CP-Net, CellViT, and NuLite achieved the best overall performance.
• *Scanner type*: The average PQ metric for each pipeline across different scanner types is presented in Fig. 4b, including Omnyx VL120 (CoNSeP), Hamamatsu Nanozoomer S60 (SegPath), 3DHistech Pannoramic Flash III (TNMI), and unknown scanner types (ILCD, Lizard, NuCLS, and PanopTILs). The variability in performance across scanner types highlights the impact of scanner type on performance. Most pipelines achieved better performance on the Omnyx VL120 scanner, which may be attributed to CoNSeP being a single-dataset benchmark, whereas the other scanner groups contain multiple datasets. Different scanners produce variations in color distribution, contrast, sharpness, noise, and artifacts, leading to domain shifts between datasets.^21^, ^22^, ^25^
• *Nuclei type*: The average PQ metric for each pipeline across different nuclei type are presented in Fig. 4c. Most pipelines achieved better performance for tumor type, followed by immune cells and then other cell types. Tumor nuclei are often easier to recognize because they are larger and morphologically distinctive, whereas immune and other cells are smaller, denser, and more heterogeneous.^18^, ^25^
• *Magnification:* The average PQ metric for each pipeline across different magnification levels is presented in Fig. 4d. Most pipelines achieved better performance at 40× compared with 20× magnification. The nuclei at 40× appear larger and contain clearer morphological and texture information, with more visible boundaries. Also, most existing pipelines were originally developed and trained primarily on 40× datasets.^18^, ^20^, ^22^
• *Performance vs efficiency*: Fig. 4e presents the trade-off between inference time and PQ for the evaluated pipelines. CP-Net, CellDETR, and CellViT form the Pareto frontier. Among them, CP-Net achieved the most favorable balance, providing high PQ performance (slightly lower than CellViT) while maintaining low inference time compared with the other evaluated pipelines. The inference times of CellViT, CP-Net, and the complete CellQuant-Net pipeline on two representative WSIs at 20× and 40× magnifications are summarized in Table 1.

**Table 1:** Runtime comparison (minutes) of CellViT, CP-Net, and the proposed CellQuant-Net on two whole-slide images of different magnifications. CellQuant-Net runtime includes WSI quality assessment, CP-Net inference, and post-processing (nuclei connectivity and statistics), with the total execution time reported.

	CellViT	CP-Net	CellQuant-Net
WSI1 [20×, 31871 × 25,199 pixels]	5.15	2.16	WSI-QA:2.67; CP-Net: 2.16; post-processing:0.94; total: 5.77
WSI2 [40×, 44125 × 70,507 pixels]	8.30	2.77	WSI-QA:12.58; CP-Net: 2.77; post-processing:1.48; total: 16.83

**Fig. 4:**
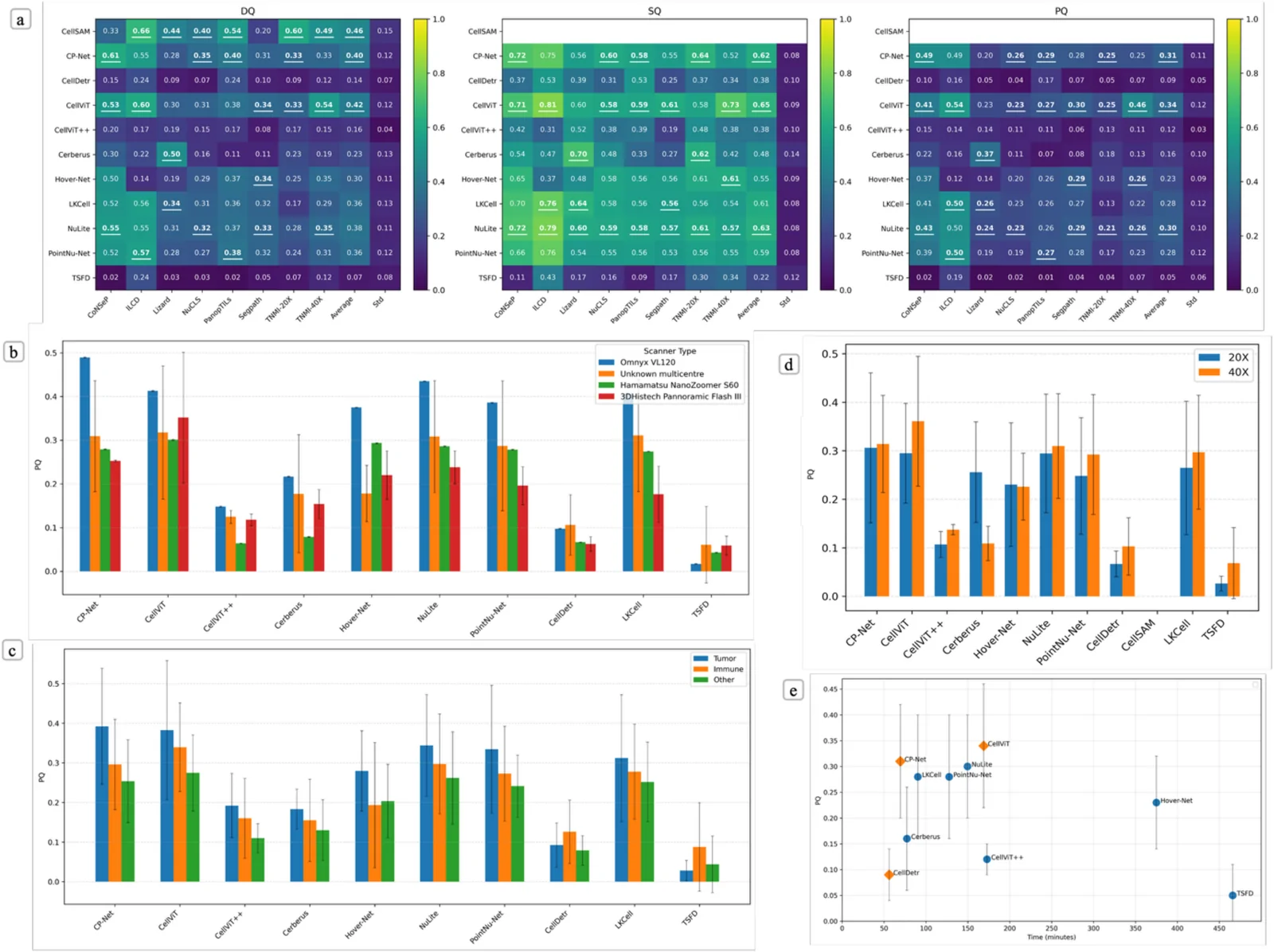
Comprehensive benchmarking of nuclei detection and classification pipelines across multiple datasets, scanner types, magnifications, and nuclei types. (a) DQ, SQ, and PQ for each pipeline the evaluated datasets, with the average performance and standard deviation shown in the last two columns. Because CellSAM only performs nuclei detection, only DQ results are available. For each dataset, the top three performing pipelines are highlighted (underlined in white). (b) Average PQ performance grouped by scanner type. (c) Class-wise PQ comparison for tumor, immune, and other nuclei type. (d) PQ comparison between 20× and 40× magnifications. (e) Trade-off between inference time and PQ (averaged over datasets) for the evaluated pipelines. Pipelines marked by orange squares, CP-Net, CellDetr, and CellViT indicate the Pareto frontier. The best trade-off between performance and computational efficiency is obtained for CP-Net.

As shown in Table S1, the Lizard dataset contained 7429 image tiles, of which 243 originated from PanNuke and 144 from CoNSeP. To assess the potential impact of source-level overlap, all PanNuke- and CoNSeP-derived tiles were excluded, and CP-Net was re-evaluated on the remaining data. Only a slight decrease in performance was observed (PQ: 0.20 → 0.18, DQ: 0.28 → 0.25, SQ: 0.56 → 0.54).

It is important to note that most available nuclei benchmark datasets do not provide WSI or patient identifiers for the released image tiles. Consequently, model performance cannot be further assessed at the WSI or patient level, and paired slide-level statistical comparisons are not feasible. Future public datasets should include WSI identifiers to enable more rigorous slide-level evaluation and statistical analysis of competing pipelines.

## 6 CellQuant-Net for nuclei quantification

Fig. 5 presents the proposed CellQuant-Net that integrates WSI QA, nuclei detection and classification, spatial neighborhood connectivity and quantification analysis. First, the QA pipeline^26^ identifies high-quality tissue regions and excludes artifacts such as irrelevant background, blurred, folded, and pen-marked regions. Next, CP-Net is applied to detect and classify nuclei. Then, the neighborhood connectivity analysis^38^ was applied to generate a spatial nuclei connectivity metric. Each nucleus is connected to its neighboring nuclei within a predefined radius. Finally, the extracted spatial connectivity information is used to compute several quantitative metrics, including cell counts, percentages, densities, and immune infiltration score. In addition, CP-Net was trained on individual datasets to provide single- and multi-organ models for downstream computational pathology analysis and AI-assisted clinical interpretation. The pipeline and pretrained models will be publicly available.

**Fig. 5:**
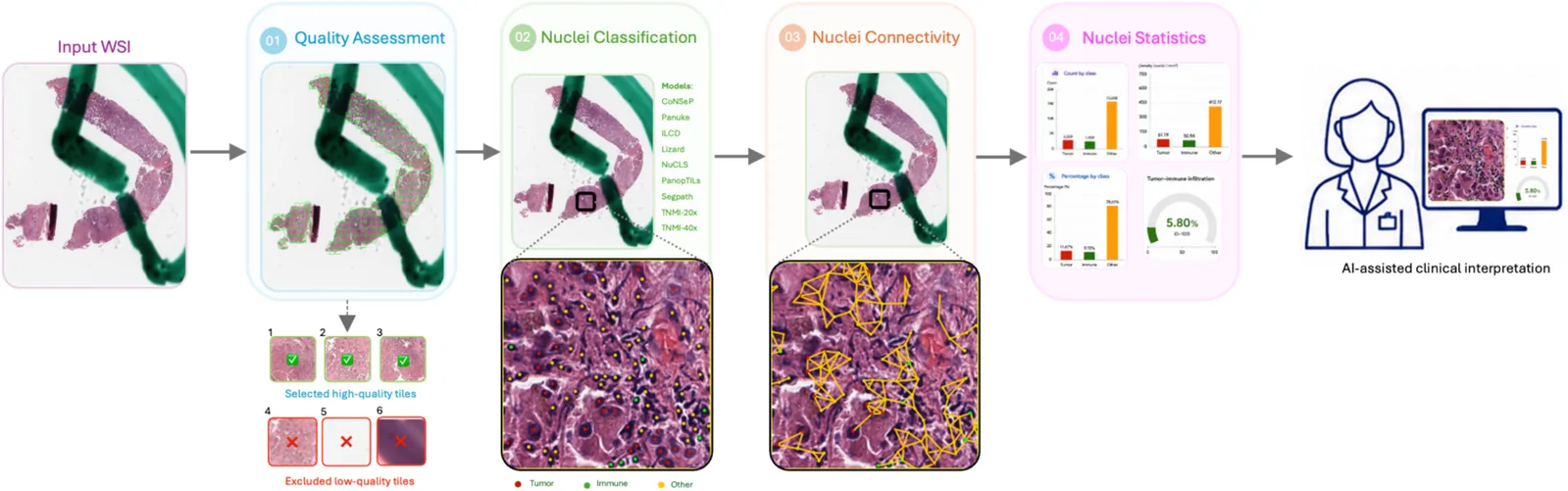
Overview of the CellQuant-Net pipeline for nuclei quantification: (1) WSI quality assessment (QA) to identify high-quality tissue tiles {1–3} and exclude tiles with artifacts {blurred:4; background:5; fold:6}; (2) nuclei detection and classification using CP-Net to identify nuclei types; (3) spatial nuclei connectivity analysis to characterize cell–cell relationships; (4) statistical analysis to compute nuclei quantification metrics.

As a proof-of-concept of its clinical applicability, CellQuant-Net was applied to 13 H&E WSIs from the AB-real hepatocellular carcinoma cohort with matched clinical annotation, deriving tumor-infiltrating lymphocyte (TIL) density for each case. Patients were stratified into high- and low-TIL groups based on the median TIL density. In this exploratory, hypothesis-generating analysis, high TIL density was associated with significantly longer overall survival (log-rank *p* = 0.006), consistent with the established prognostic role of lymphocytic infiltration in HCC (Supplementary materials: Table S3, Fig. S1, Fig. S2). Given the limited sample size, these preliminary findings should be interpreted with caution and are not intended as a clinical validation; they nonetheless illustrate the pipeline's potential to generate biologically and clinically meaningful readouts from routine H&E images.

## 7 Conclusion and future work

This article presents CP-Net for H&E-WSI nuclei detection and classification. By incorporating H-based prior information, CP-Net guides the network toward nuclei-relevant regions while maintaining a lightweight architecture and fast inference speed. Extensive benchmarking on eight public and private datasets obtained from different scanners, organs, magnifications, and clinical centers demonstrated that CP-Net achieves competitive performance compared with state-of-the-art pipelines while maintaining high computational efficiency. The results also indicate that organ type, scanner type, cell type, tissue composition, and magnification can substantially influence model performance.

In addition, CellQuant-Net is introduced, an end-to-end nuclei quantification pipeline that integrates WSI QA, nuclei detection and classification, and spatial neighborhood analysis. The pipeline allows the extraction of several quantitative nuclei metrics, facilitating downstream computational pathology analysis.

Although CP-Net achieved competitive performance, several limitations and aspects for enhancement remain. Future work will therefore focus on the following directions:

• Collect additional multi-center datasets encompassing different organs, scanner types, staining procedures, magnifications, cancer grades, and histological subtypes to support the development, training, testing, and standardized cross-dataset benchmarking of nuclei detection and classification pipelines under a unified evaluation framework. Such standardized training and evaluation protocols will improve model robustness, reproducibility, and generalizability while enabling more comprehensive and equitable comparisons across different model architectures. In addition, evaluating all methods under a common training protocol would eliminate potential biases introduced by differences in annotation schemes, such as class merging, and provide fairer benchmarking of state-of-the-art approaches. Ultimately, these efforts could facilitate the development of pan-cancer models or, more realistically, models that generalize across common histological subtypes (e.g., adenocarcinoma) in major solid tumors, including breast, prostate, colorectal, and lung cancers.
• Extend benchmarking beyond detection and classification metrics by exploring the association between cell quantification results with independent biological measurements obtained from orthogonal modalities, including IHC and RNA expression data, to further evaluate the biological relevance and clinical utility of the proposed pipeline.
• Extend CP-Net to support a broader range of nuclei categories by leveraging immunofluorescence (IF)-guided annotations and co-registered H&E–IF datasets, enabling more accurate cell-type labeling and improved nuclei detection and classification performance.
• Use advanced 3D pathology imaging to move beyond 2D analysis toward 3D nuclei detection and classification, enabling more accurate characterization of nuclei morphology, while providing richer features to further improve model performance.

## Ethics statement

This study used internal datasets, for which all patients provided informed consent and data collection was approved by the Institutional Review Board at each participating institute (ILCD: R16008; TNM-I: TNM-I-REK2016/2054), as well as publicly available anonymized datasets. Therefore, no additional ethical approval or informed consent was required, and all data were used in accordance with the terms set by the original data providers.

## Funding

This research is supported by the 10.13039/501100007137Northern Norway Regional Health Authority (grant HNF1771–26 and HNF1660–23). The funders had no role in the design and conduct of the study, nor in the decision to submit the manuscript for publication.

## Declaration of generative AI

During the preparation of the first draft of this manuscript, the corresponding author used ChatGPT-4o for grammar check and readability of the text. Afterwards, the manuscript was reviewed and edited by the co-authors as needed. The corresponding author then performed a final review and editing of the manuscript, taking full responsibility for the content of the published article. Generative AI was not used to write the content.

## Declaration of competing interest

Dr. Pinato reported lecture fees from 10.13039/501100000801Bayer Healthcare, 10.13039/100004325AstraZeneca, 10.13039/501100003769EISAI, 10.13039/100008021Bristol Myers Squibb, 10.13039/100004337Roche, 10.13039/100008497Boston Scientific, and Ipsen; travel support from 10.13039/100008021Bristol Myers Squibb, 10.13039/100004337Roche, 10.13039/501100000801Bayer Healthcare, and 10.13039/100004325AstraZeneca; and consulting fees from Accession Therapeutics, MAIA Therapeutics, 10.13039/100008021Bristol Myers Squibb, Mina Therapeutics, Galapagos, Parabilis, 10.13039/100001003Boehringer Ingelheim, Ewopharma, 10.13039/501100003769EISAI, Ipsen, 10.13039/100004337Roche, H3 Biomedicine, 10.13039/100004325AstraZeneca, DaVolterra, Mursla, 10.13039/501100008645Terumo, Avammune Therapeutics, Starpharma, LiFT Biosciences, and Exact Sciences. All outside the submitted work. Dr. Rakaee reported honoraria from AstraZeneca. No other COIs were reported.

## Data Availability

The annotated image tiles for the TNMI and ILCD datasets will be made available for research purposes upon request. The remaining datasets used in this study are publicly available: PanNuke, CoNSeP, Lizard, NuCLS, PanopTILs, and SegPath. The source code for the proposed pipeline is publicly available on GitHub.

## Appendix A Supplementary data

Supplementary material Dataset details (Table S1) and benchmarked pipeline architectures (Table S2); TIL density and clinical outcomes for the 13-patient HCC cohort (Table S3) with corresponding overall-survival and progression-free-survival Kaplan-Meier curves (Fig. S1, S2); qualitative ground-truth vs. predicted nuclei comparisons across datasets and pipelines (Table S4).
