# Supplementary material for: CellPrior-net: Prior-guided nuclei detection and classification for H&E whole-slide images
Source: J Pathol Inform. 2026 Aug 31;23:100716. doi: 10.1016/j.jpi.2026.100716 (PMC13636067; doi:10.1016/j.jpi.2026.100716)
Supplement: Supplementary file 1 [file mmc1.pdf]

# Supplementary Materials

**ConSeP** [1]: This dataset consists of 41 image tiles collected from colon cancer patients at the University Hospitals Coventry and Warwickshire, UK. The original tile size is 1000 × 1000 pixels with a spatial resolution of 0.25 µm/px at 40× magnification. The annotations were obtained by a pathologist and subsequently reviewed by a second pathologist to achieve consensus. The dataset contains multiple nuclei categories, including inflammatory, epithelial, fibroblast, muscle, and endothelial cells.

**ILCD**: This dataset consists of 19,016 image tiles collected from 40 WSIs of liver cancer patients at Imperial College London across multiple centers. Each tile has a size of 256 × 256 pixels with a spatial resolution of 0.25 µm/px at 40× magnification. The dataset contains three cell categories: immune cells, tumor cells, and other cells. The annotations were initially performed by two PhD students and one postdoctoral researcher and were subsequently reviewed by a pathologist to ensure annotation quality and accuracy.

**Lizard** [2]: This dataset extends the CoNSeP dataset by integrating colon cancer tissue images collected from multiple centers in the USA and four hospitals in China. The dataset has a spatial resolution of 0.50 µm/px using 20× magnification and contains 270 image tiles of varying sizes, with an average dimension of 1016 × 917 pixels. The annotations were generated through automatic, semi-automatic, and manual boundary refinement procedures. The dataset contains multiple nuclei categories, including neutrophils, lymphocytes, plasma cells, eosinophils, epithelial cells, and connective tissue cells.

**NuCLS** [3]: This dataset is a crowdsourced collection of nuclei annotations from breast cancer tissue obtained from TCGA, annotated by medical students and pathologists. The dataset was acquired at a spatial resolution of 0.20 µm/px using 20× magnification and contains 151 large image tiles of varying sizes, with an average dimension of 3300 × 2500 pixels. The dataset includes multiple tissue and cellular categories, including tumor, stroma, lymphocytic infiltrate, necrosis or debris, glandular secretions, blood, metaplasia NOS, fat, plasma cells, other immune infiltrate, mucoid material, normal acinus or duct, lymphatics, nerve, skin adnexa, blood vessel, angioinvasion, ductal carcinoma in situ (DCIS), and other tissue regions.

**PanopTILs** [4]: This dataset provides region and cell-level annotations obtained from breast cancer patients within the TCGA cohort. The dataset was acquired at a spatial resolution of 0.25 µm/px using 40× magnification and consists of 157 large image tiles, each 1024 × 1024 pixels. It includes pathologist-approved nuclei annotations for multiple cell types, including TILs, stromal cells, epithelial cells, miscellaneous cells, plasma, and other.

**SegPath** [5]**:** This dataset consists of 158,687 registered H&E and immunofluorescence (IF) image patches collected from 18 organs at the University of Tokyo Hospital. The images have a size of 984 × 984 pixels with a spatial resolution of 0.22 µm/px at 40× magnification. The annotations were automatically generated through IF-based registration rather than manual pathologist annotation. The dataset provides region-wise segmentation masks for epithelium (Pan-cytokeratin), smooth muscle/myofibroblast (αSMA), lymphocytes (CD3/CD20), leukocytes (CD45RB), blood/lymphatic vessels (ERG), plasma cells (MIST1), myeloid cells (MNDA), and red blood cells (CD235a). This dataset differs substantially from conventional nuclei detection and classification datasets, as it does not provide manually annotated cell-instance segmentations. Instead, it contains registered H&E and immunofluorescence (IF) images, along with region-wise semantic segmentation masks generated automatically from the IF staining. Following the methodology described in CellViT++ [6], cell-level annotations were derived from the registered H&E and immunofluorescence (IF) images. Specifically, CellViT was first applied to the H&E patches to detect and segment individual nuclei. The resulting instance segmentation masks were then mapped onto the registered IF semantic masks to assign cell identities. This procedure enables the automatic generation of large-scale cell-level datasets from registered H&E and IF images without requiring manual cell-instance annotation.

**PanNuke** [7]: This dataset consists of 7904 image tiles collected from 19 different organs. The images have a size of 256 × 256 pixels with a spatial resolution of 0.25 µm/px at 40× magnification. The dataset contains annotated nuclei categorized into five major cell types, including neoplastic, inflammatory, connective tissue, dead, and epithelial cells.

**TNMI** [8]: This dataset consists of 31,742 image tiles at 20× magnification and 18,424 image tiles at 40× magnification collected from lung cancer patients enrolled in the Scandinavian multi-institutional TNM-I clinical trial. The images have a size of 256 × 256 pixels with a spatial resolution of 0.50 µm/px at 20× magnification and 0.25 µm/px at 40× magnification. The dataset contains annotated nuclei categorized into three major cell types, including tumor, immune, and other cells.

**Table S1**. Summary of the datasets used in this study, including the number of image tiles, distribution of annotated cell types (tumor, immune, and other), total number of annotated cells, image magnification, scanner type, and organ. The datasets cover both single-organ and multi-organ histopathology cohorts obtained using different scanners and magnification settings.

| Dataset | No. of Tiles | No. of Tumor | No. of Immune | No. of Other | Total No. of Cells | Magnification | Scanner Type | Organ |
|---|---|---|---|---|---|---|---|---|
| **ConSeP** | 983 | 14310 | 9291 | 16512 | 40113 | 40x | Omnyx VL120 | Colon |
| **ILCD** | 19016 | 120979 | 195921 | 39412 | 356312 | 40x | Unknown-multicenter | Liver |
| **Lizard** | 7429 | 389722 | 226541 | 178071 | 794334 | 20x | Unknown-multicenter | Colon |
| **NuCLS** | 30464 | 760245 | 356774 | 422780 | 1539799 | 20x | Unknown-multicenter | Breast |
| **PanopTILs** | 1743 | 22244 | 9519 | 20592 | 52355 | 40x | Unknown-multicenter | Breast |
| **SegPath** | 542238 | 1837707 | 934981 | 858400 | 3631088 | 40x | Hamamatsu Nanozoomer S60 | Multi-organ |
| **PanNuke** | 7904 | 85279 | 28555 | 49137 | 162971 | 40x | Unknown-multicenter | Multi-organ |
| **TNMI-20** | 31742 | 1974156 | 276707 | 1025019 | 3275882 | 20x | 3DHistech Pannoramic Flash III | Lung |
| **TNMI-40** | 18424 | 403213 | 25156 | 119071 | 547440 | 40x | 3DHistech Pannoramic Flash III | Lung |

**Table S2**. Comparison of state-of-the-art nuclei detection and classification pipelines in digital pathology, including architecture type, model size, and supported tasks.

| Pipeline | CP-Net | CellViT | CellViT++ | Cerberus | HoverNet | NuLite | PointNu-Net | CellDetr | CellSam | LKCell | TSFD |
|---|---|---|---|---|---|---|---|---|---|---|---|
| **Parameters** | 46.4 M | 699.7 M | 699.7 M | 28.4 M | 37.6 | 47.9 | 161.4 | 207.9 | 289.6 | 163.8 | 22.1 |
| **Architecture** | CNN | ViT | ViT | CNN | CNN | CNN | CNN | ViT | ViT | CNN | CNN |
| **Task** | Detection & classification | Detection & classification | Detection & classification | Detection & classification | Detection & classification | Detection & classification | Detection & classification | Detection & classification | Detection | Detection & classification | Detection & classification |

**Table S3**.Tumor-infiltrating lymphocyte (TIL) density derived from CellQuant-Net for 13 H&E WSIs from the AB-real hepatocellular carcinoma cohort with complete matched clinical annotation. Patients were stratified into high- and low-TIL groups using the median TIL density as cut-off. CR, complete response; DCR, disease control rate; PD, progressive disease; PFS, progression-free survival; PR, partial response; OS, overall survival; SD, stable disease.

| Patient ID | TIL density (cells/mm²) | TIL group | BCLC stage | Child-Pugh score | OS (months) | PFS (months) | DCR (0/1) | Best response (RECIST) |
|---|---|---|---|---|---|---|---|---|
| Patient 1 | 845.0 | High_TIL | B | 5 | 16.3 | 2.01 | No | PD |
| Patient 2 | 245.0 | Low_TIL | C | 5 | 18.1 | 25.3 | No | PD |
| Patient 3 | 811.0 | High_TIL | C | 5 | 17.5 | 17.5 | Yes | SD |
| Patient 4 | 344.0 | Low_TIL | A | 5 | 3.09 | 3.09 | Yes | PR |
| Patient 5 | 797.0 | High_TIL | B | 6 | 18.3 | 18.3 | Yes | CR |
| Patient 6 | 1942.0 | High_TIL | C | 5 | 4.11 | 4.11 | Yes | SD |
| Patient 7 | 700.0 | High_TIL | B | 5 | 8.68 | 8.68 | Yes | PR |
| Patient 8 | 416.0 | Low_TIL | C | 5 | 6.11 | 6.11 | Yes | SD |
| Patient 9 | 989.0 | High_TIL | B | 6 | 19.2 | 16.6 | Yes | PR |
| Patient 10 | 782.0 | High_TIL | C | 5 | 20.9 | 15.0 | Yes | SD |
| Patient 11 | 86.3 | Low_TIL | C | 5 | 16.0 | 8.25 | Yes | SD |
| Patient 12 | 2.44 | Low_TIL | C | 5 | 1.64 | 0.756 | No | PD |
| Patient 13 | 134.0 | Low_TIL | B | 5 | 4.01 | 1.71 | No | PD |

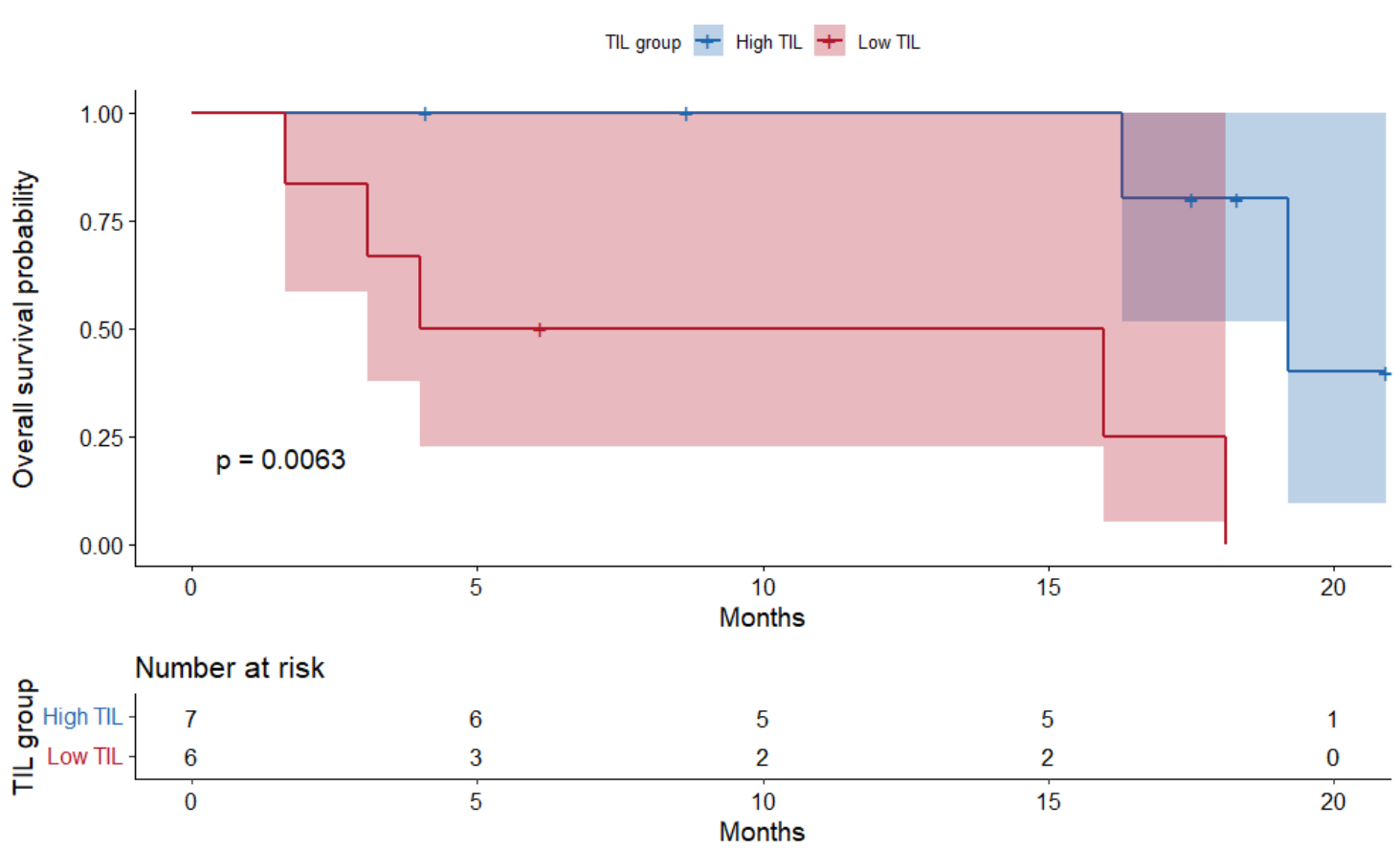


**Fig.S1**. Kaplan–Meier estimates of overall survival stratified by CellQuant-Net-derived TIL group (high vs low, split by median TIL density) in the AB-real HCC cohort (n=13). High-TIL patients showed significantly longer overall survival than low-TIL patients (log-rank p=0.006; Cox HR 11.8, 95% CI 1.3–105.1 for low- vs high-TIL).

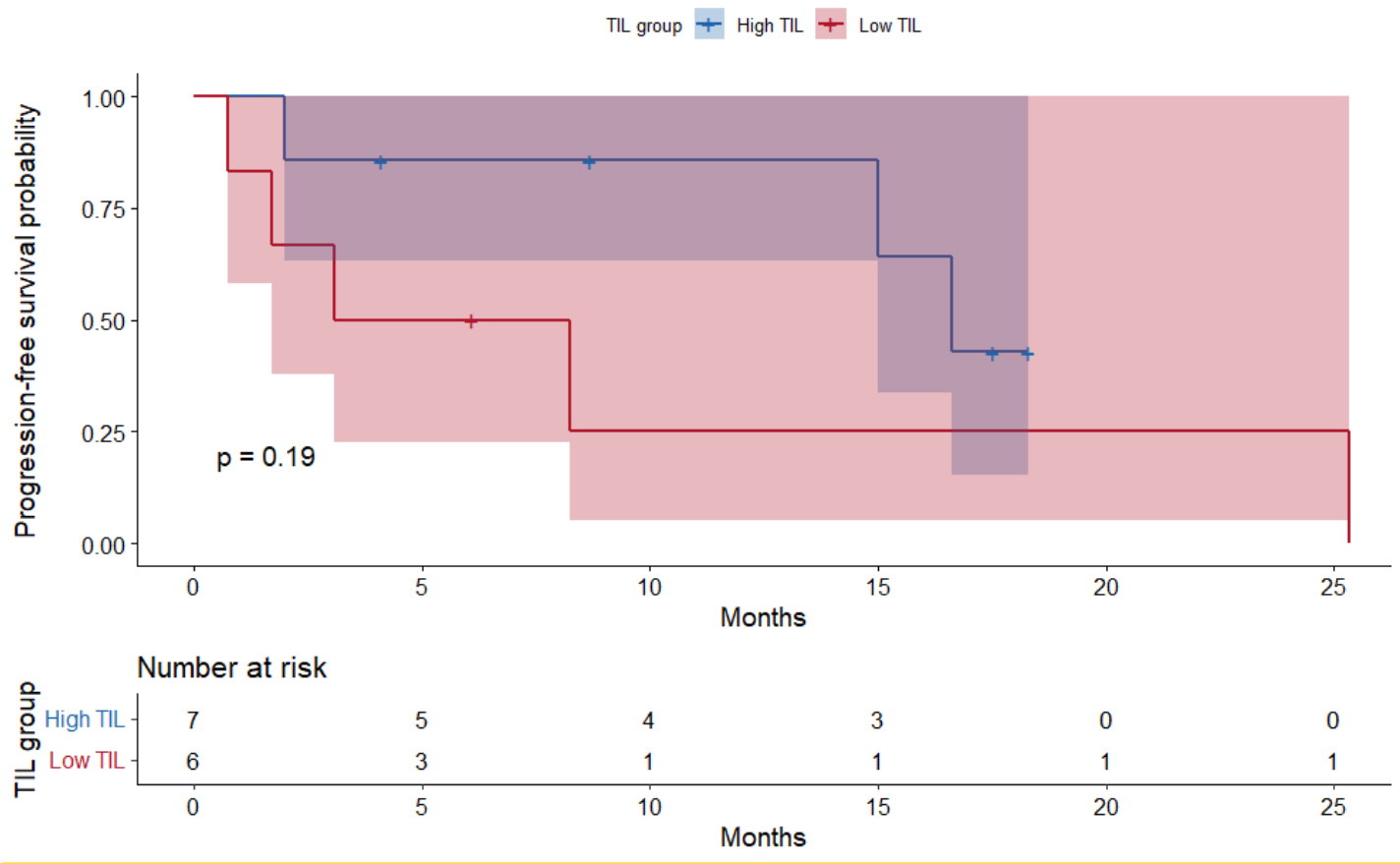


**Fig. S2**. Kaplan–Meier estimates of progression-free survival stratified by CellQuant-Net-derived TIL group (high vs low, split by median TIL density) in the AB-real HCC cohort (n=13). Progression-free survival did not differ significantly between groups (log-rank p=0.2; Cox HR 2.70, 95% CI 0.58–12.44 for low- vs high-TIL), with most patients in both groups progressing during follow-up.

**Table S4**. Comparing the ground truth (GT) annotations with predictions generated by different state-of-the-art pipelines for each dataset.

| | **CoNSeP** |
|---|---|
| | Image Tile 1 · Image Tile 2 · Image Tile 3 · Image Tile 4 |
| GT | 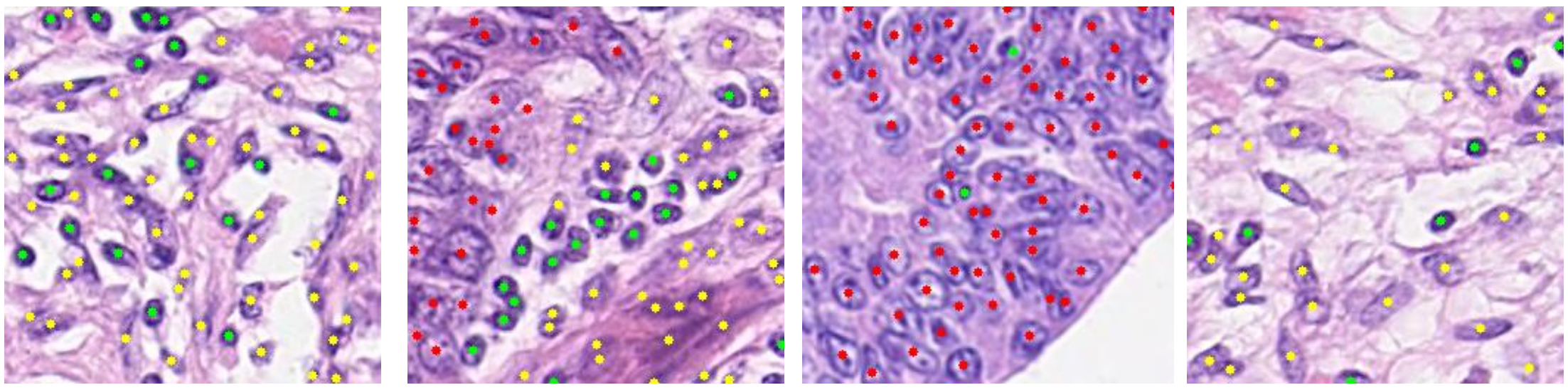 |
| CellViT | 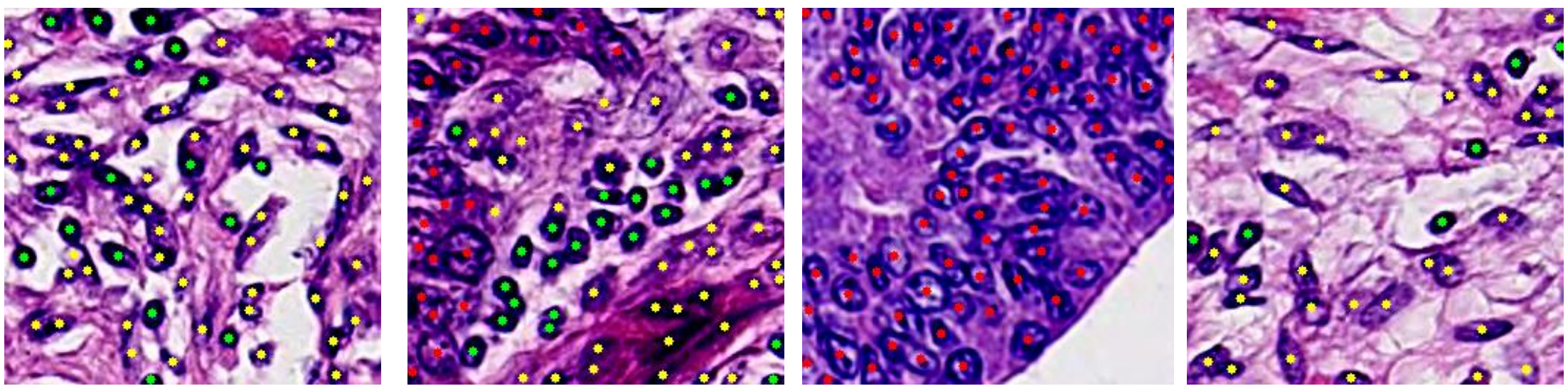 |
| CP-Net | 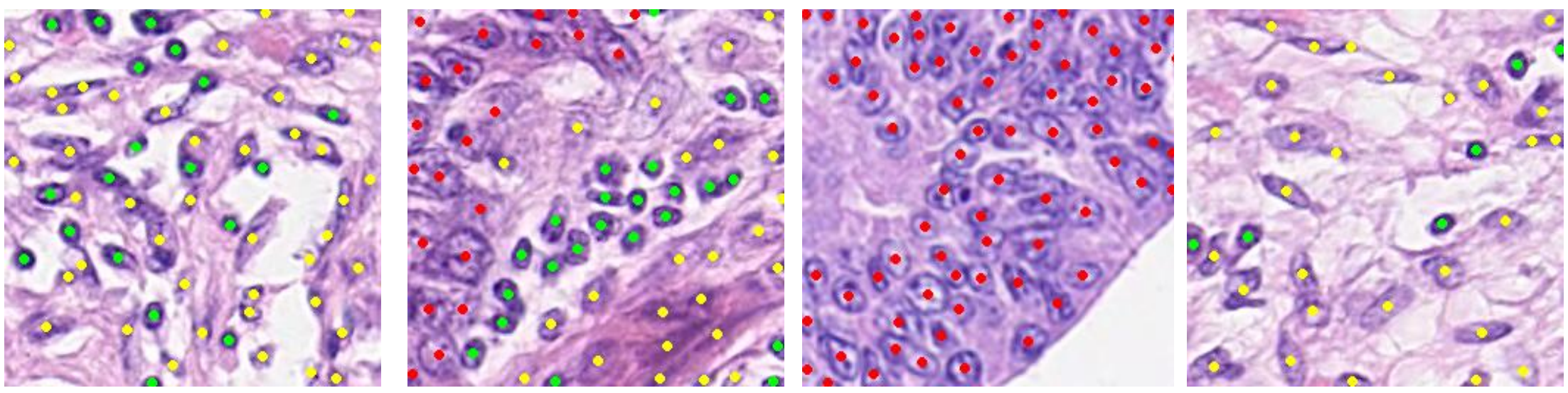 |

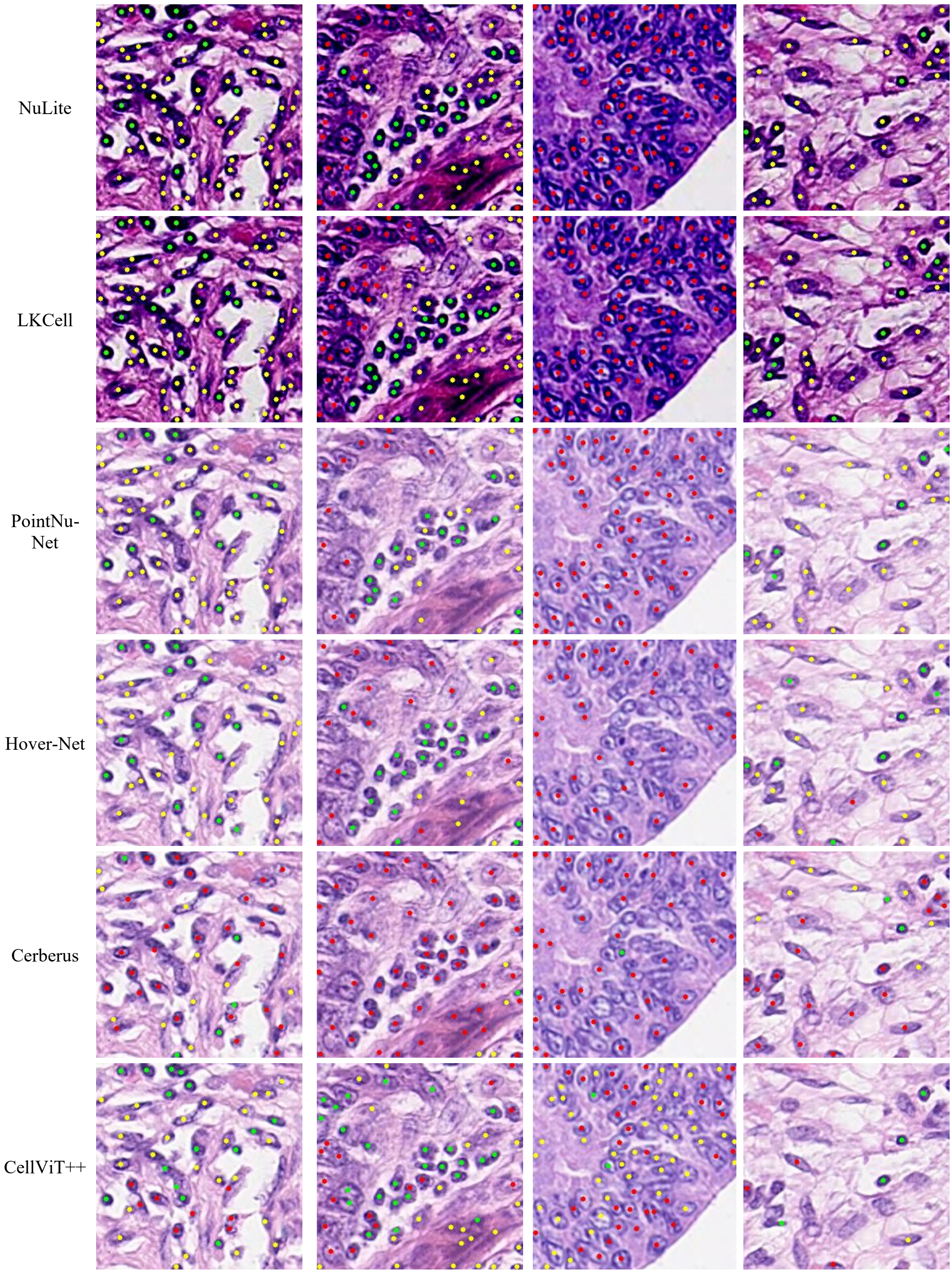
NuLite
LKCell
PointNu-Net
Hover-Net
Cerberus
CellViT++

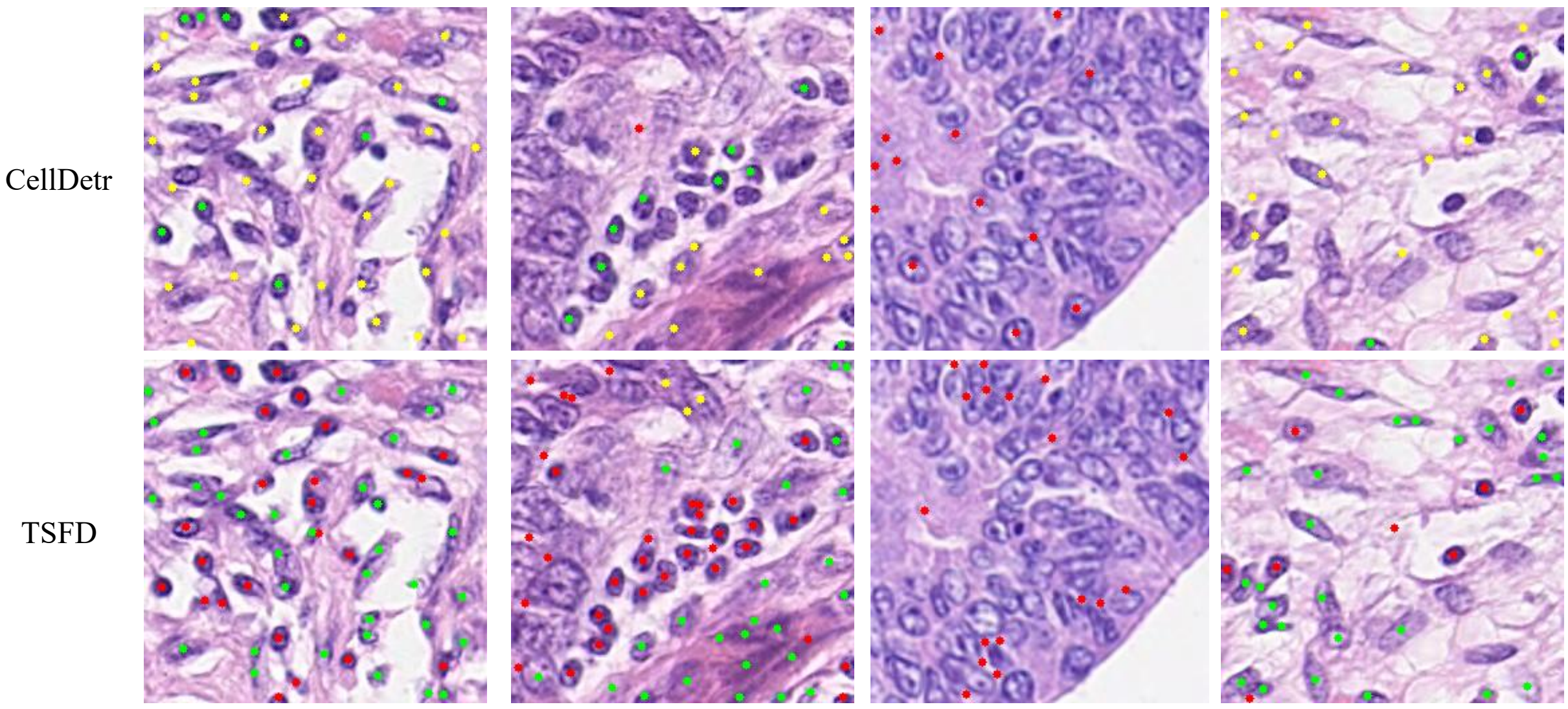
CellDetr
TSFD

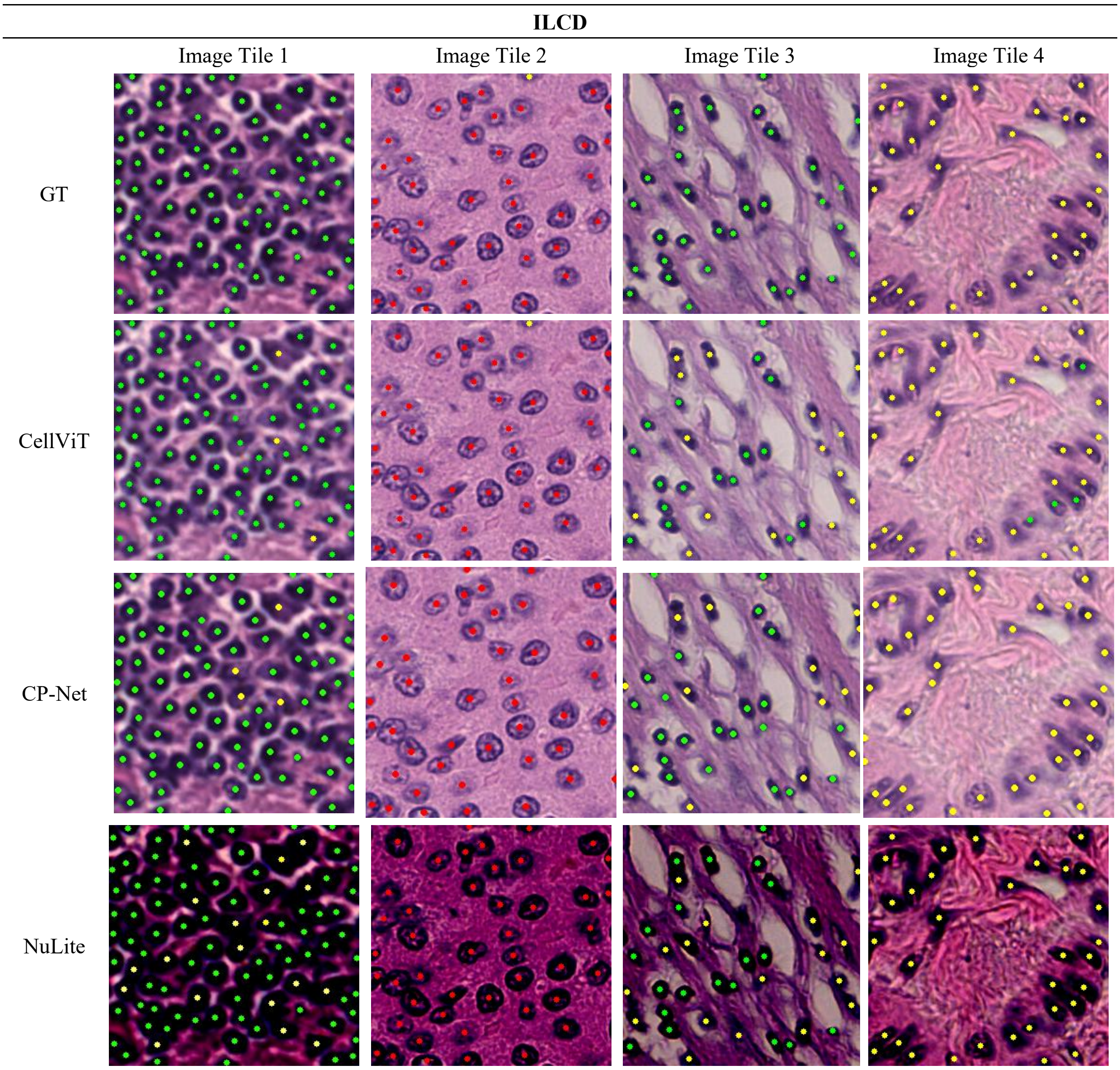
ILCD
Image Tile 1
Image Tile 2
Image Tile 3
Image Tile 4
GT
CellViT
CP-Net
NuLite

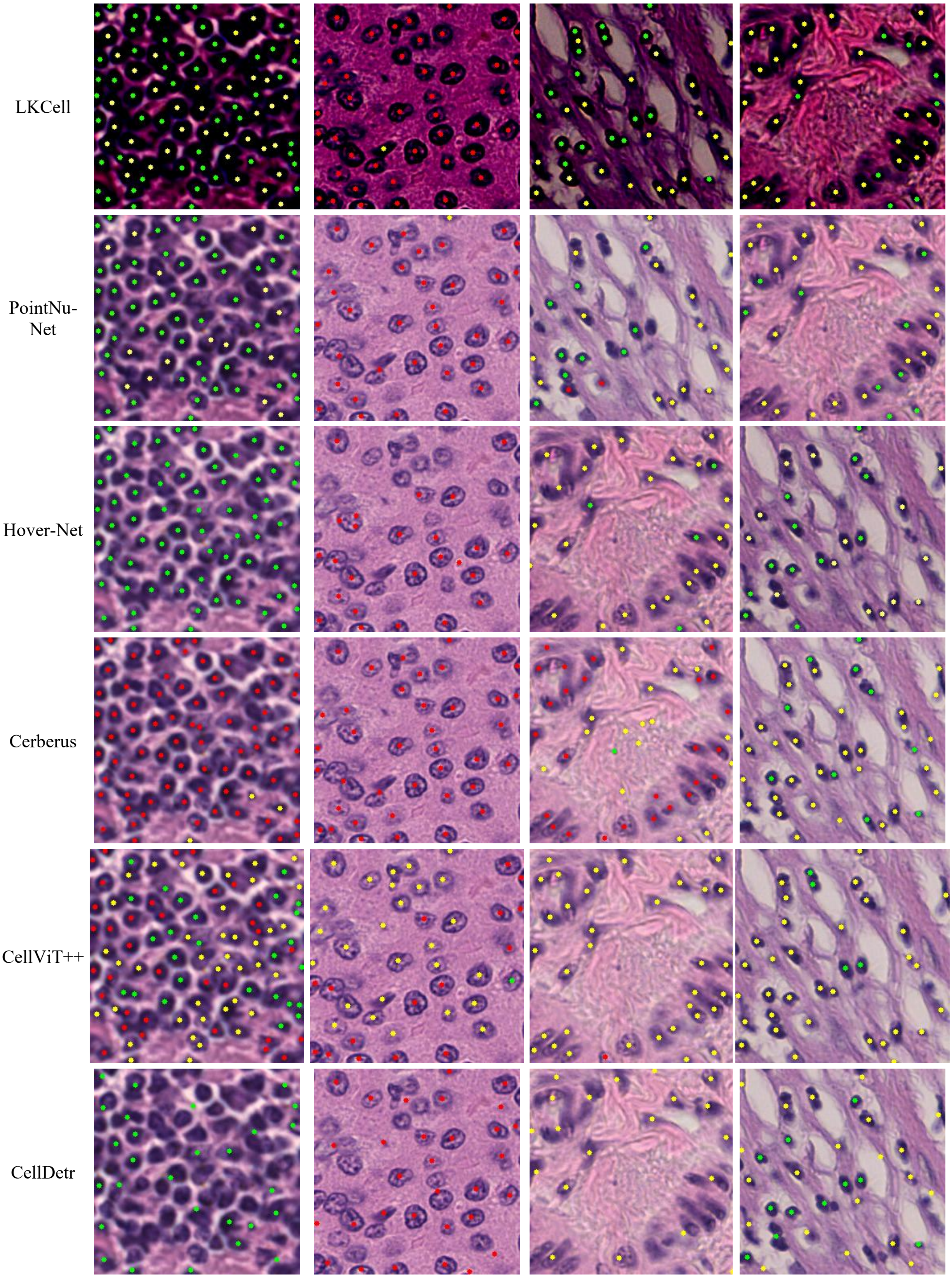
LKCell
PointNu-Net
Hover-Net
Cerberus
CellViT++
CellDetr

| | |
|---|---|
| TSFD | 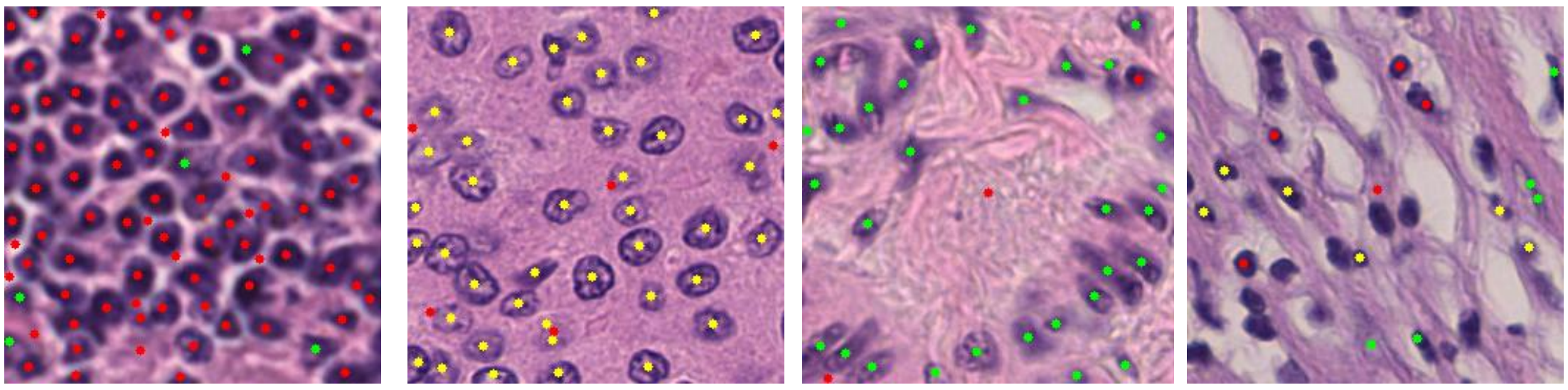 |

| Lizard | | | | |
|---|---|---|---|---|
| | Image Tile 1 | Image Tile 2 | Image Tile 3 | Image Tile 4 |
| GT | 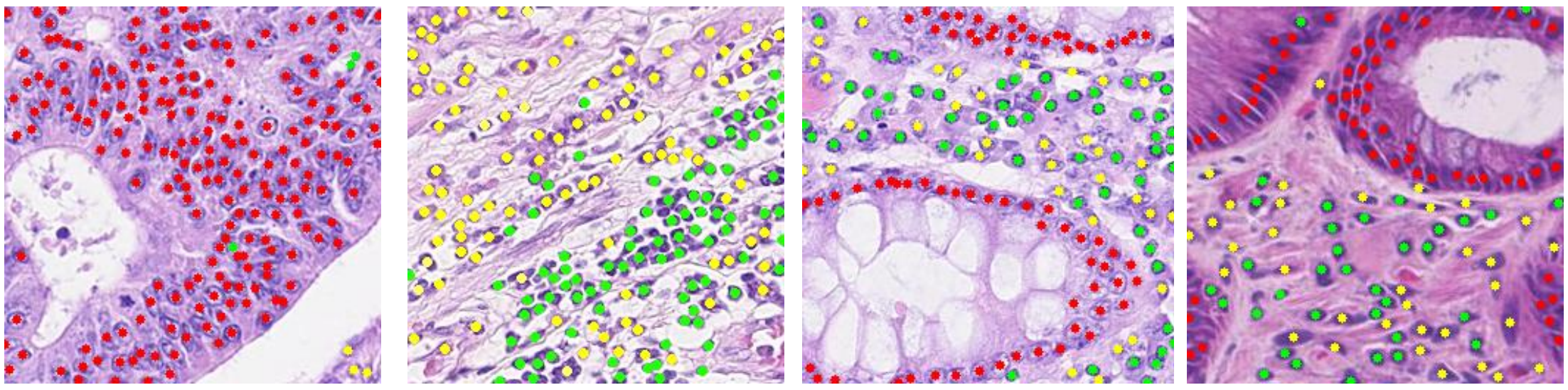 | | | |
| CellViT | 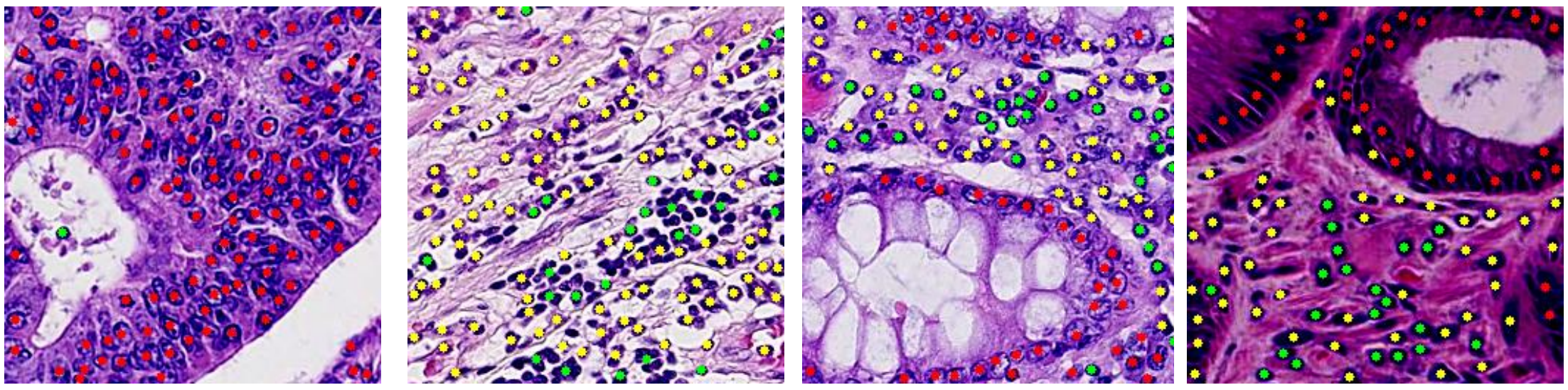 | | | |
| CP-Net | 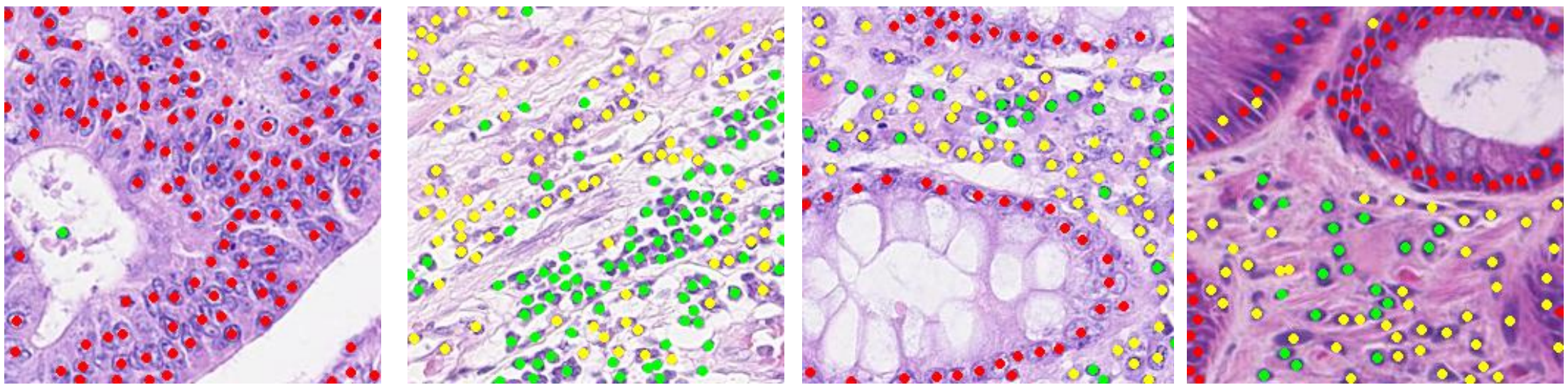 | | | |
| NuLite | 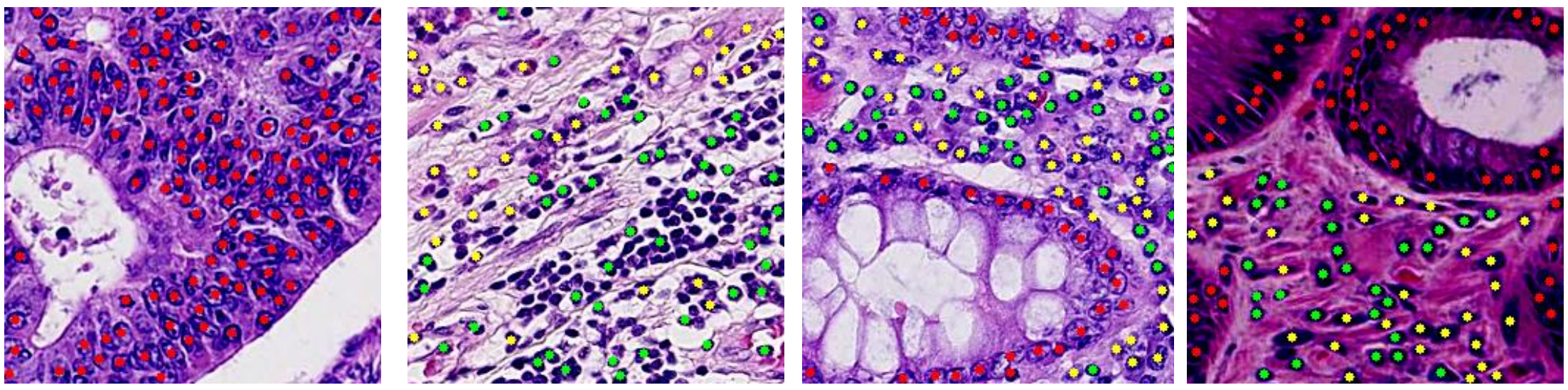 | | | |

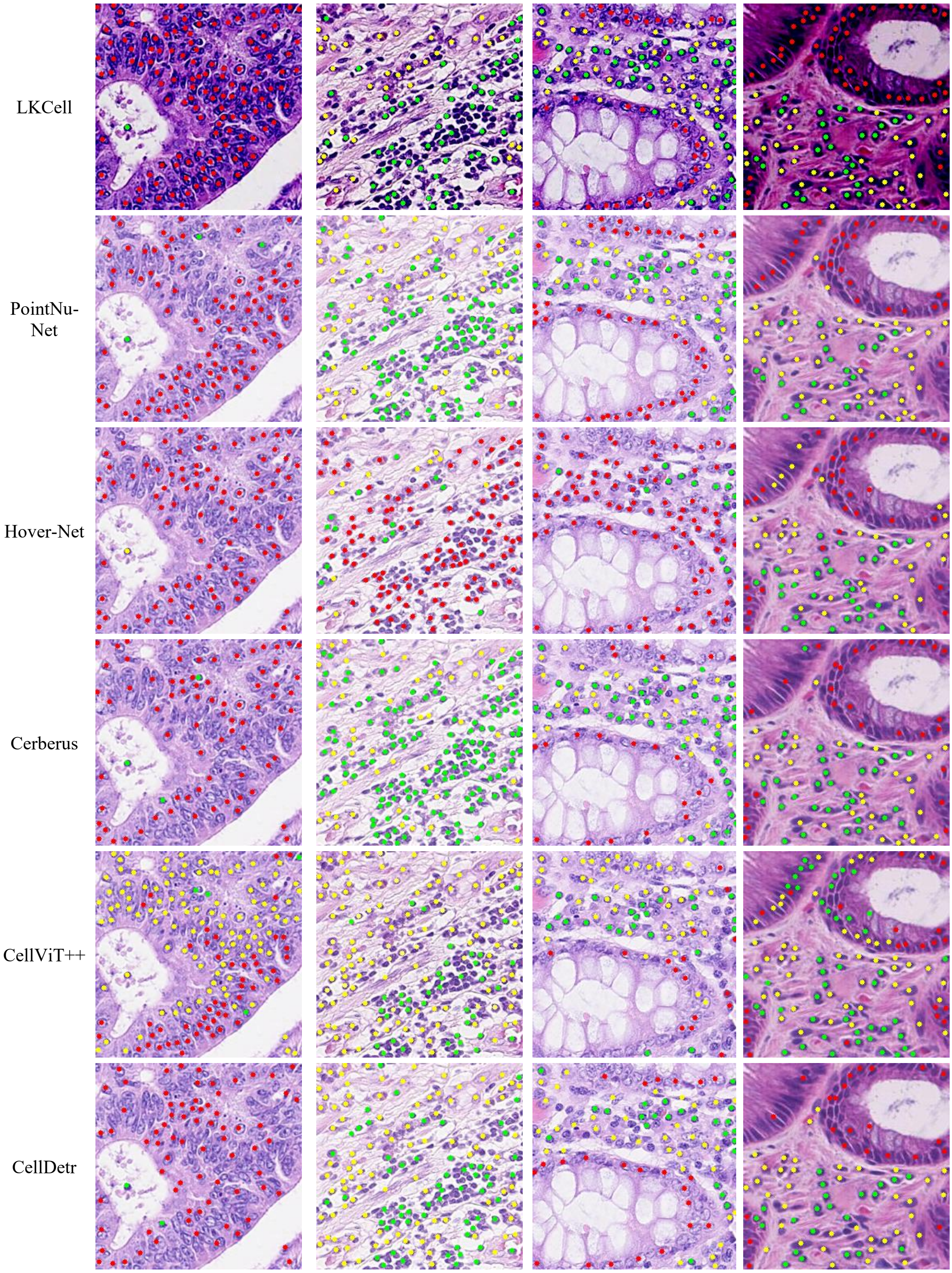
LKCell
PointNu-Net
Hover-Net
Cerberus
CellViT++
CellDetr

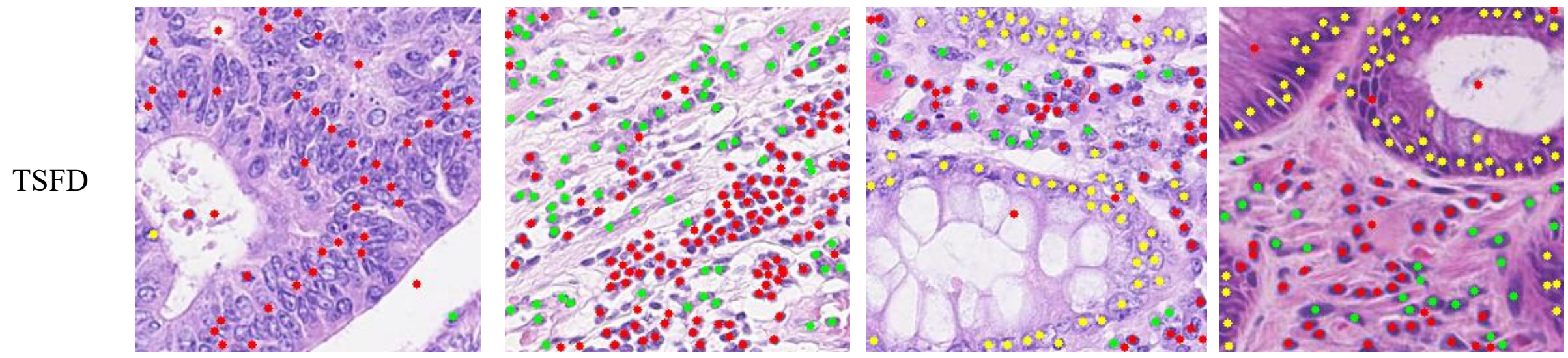
TSFD

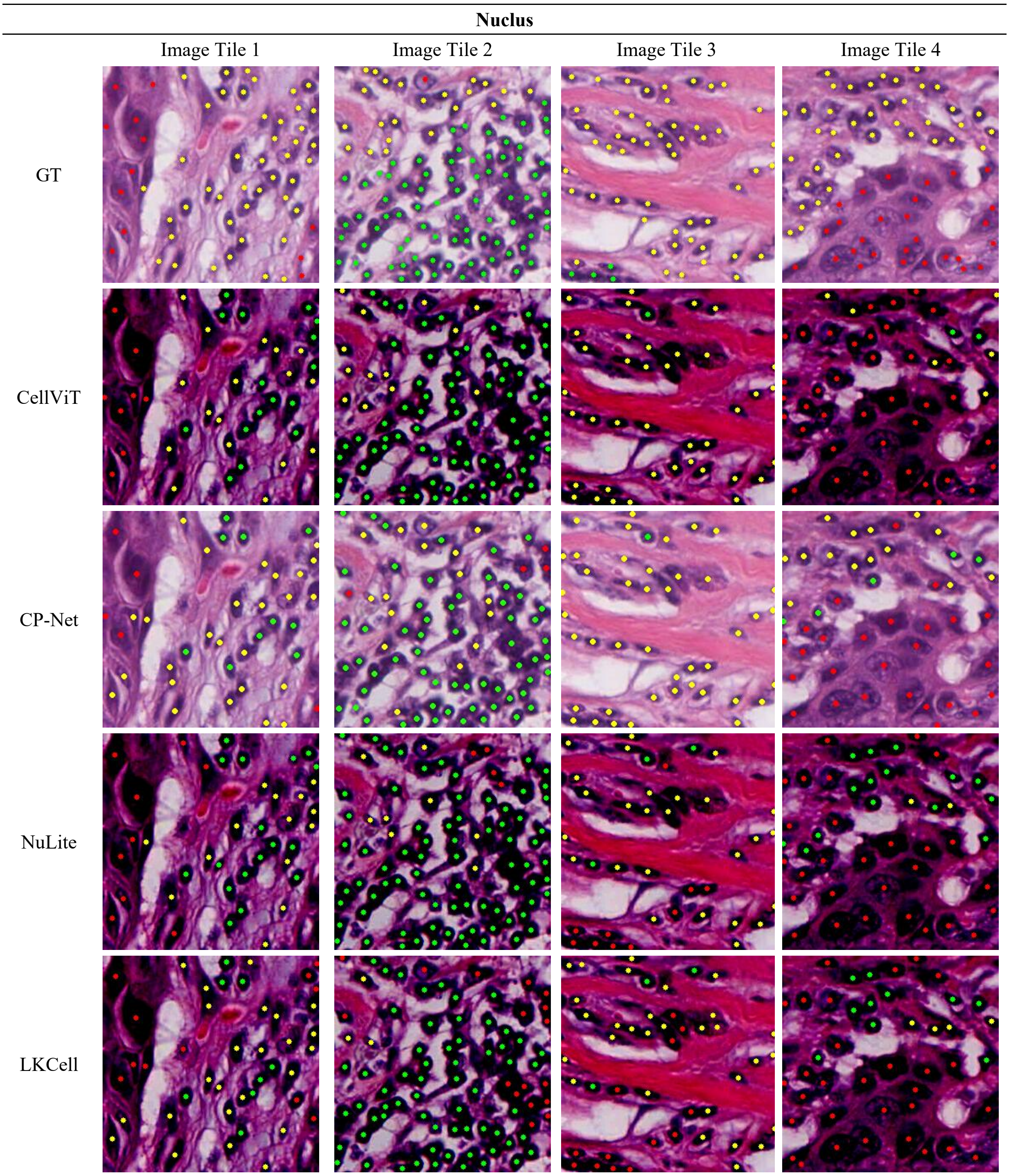
Nuclus
Image Tile 1
Image Tile 2
Image Tile 3
Image Tile 4
GT
CellViT
CP-Net
NuLite
LKCell

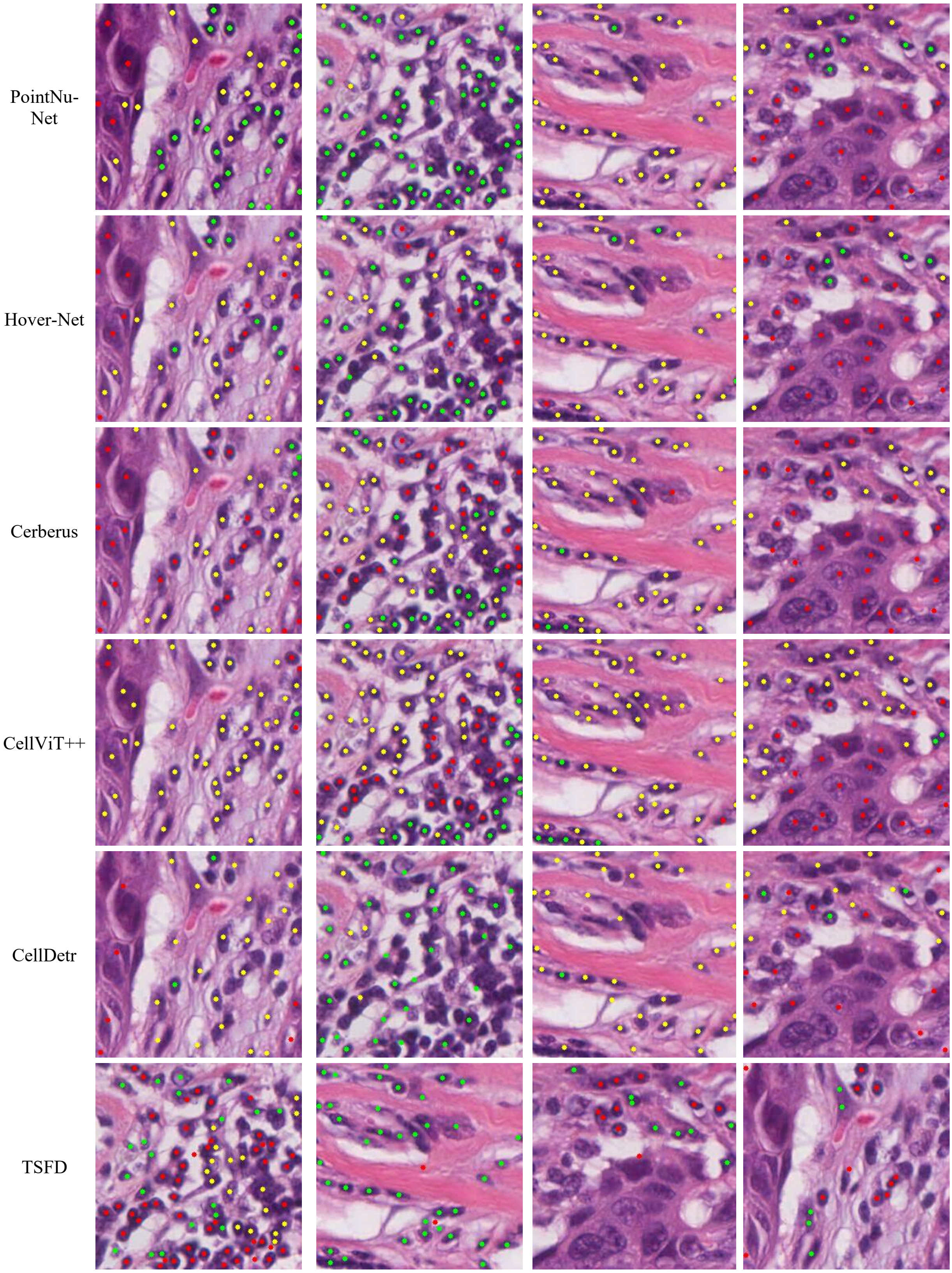
PointNu-Net
Hover-Net
Cerberus
CellViT++
CellDetr
TSFD

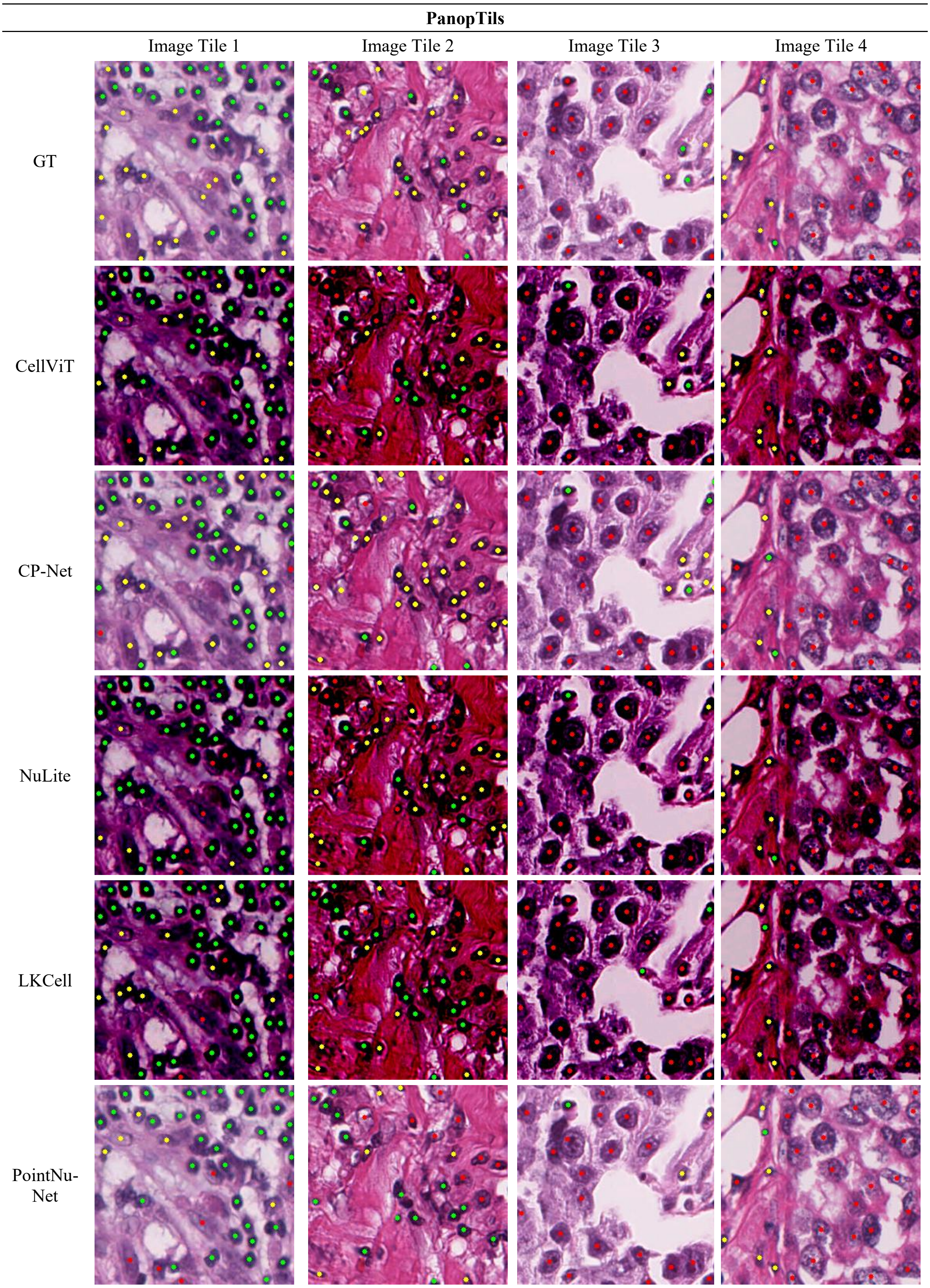
PanopTils
Image Tile 1
Image Tile 2
Image Tile 3
Image Tile 4
GT
CellViT
CP-Net
NuLite
LKCell
PointNu-Net

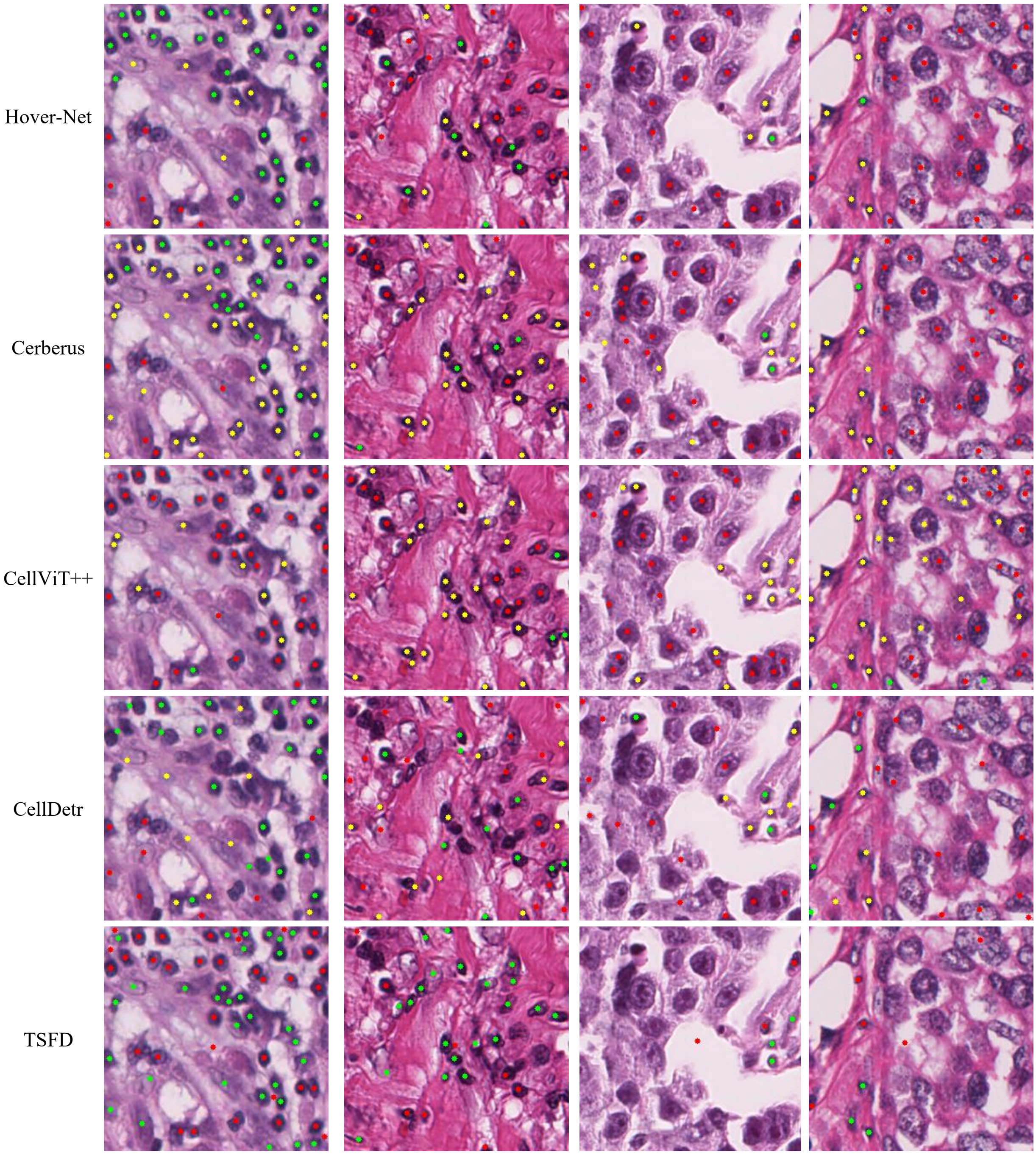

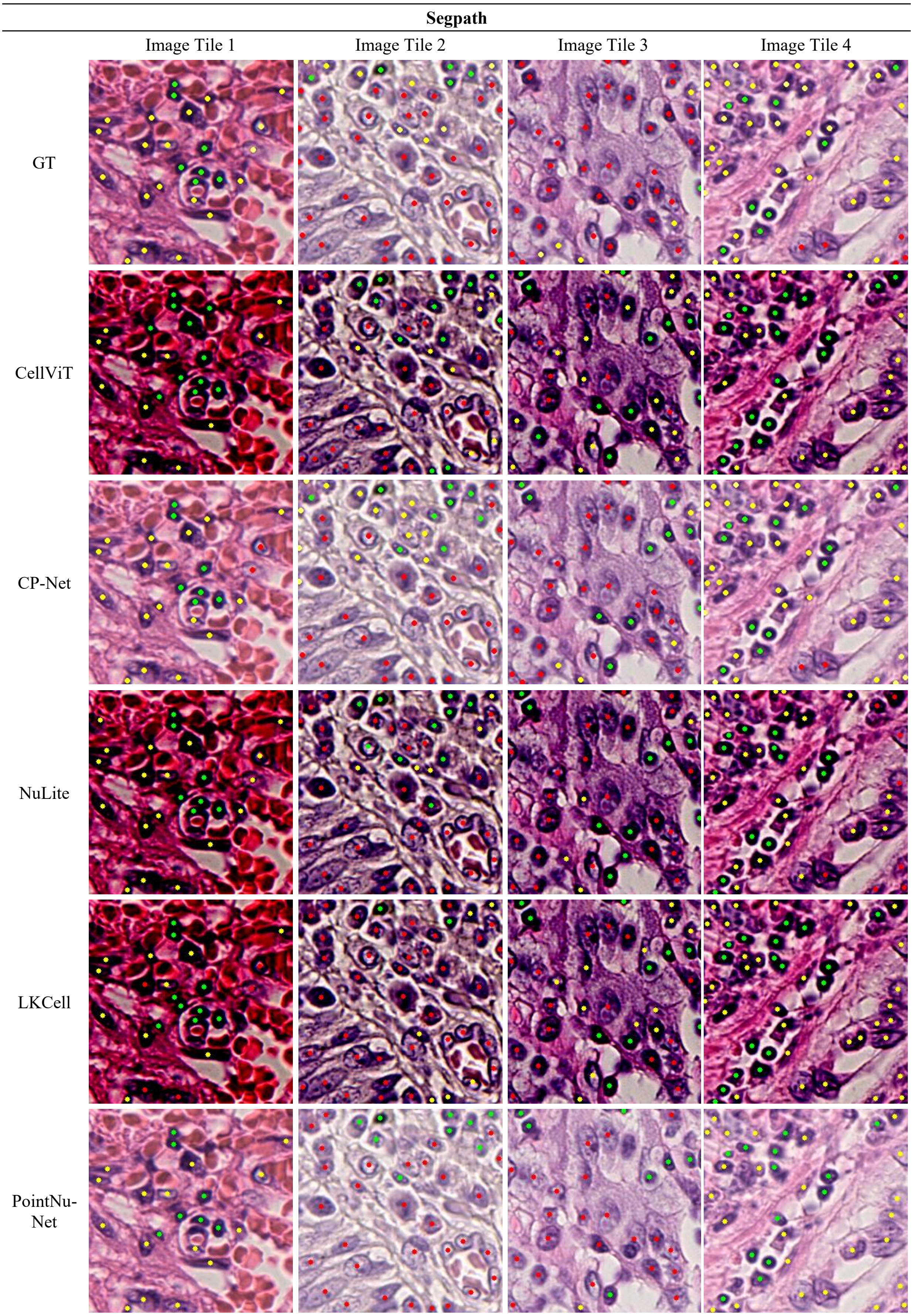
Segpath
Image Tile 1
Image Tile 2
Image Tile 3
Image Tile 4
GT
CellViT
CP-Net
NuLite
LKCell
PointNu-Net

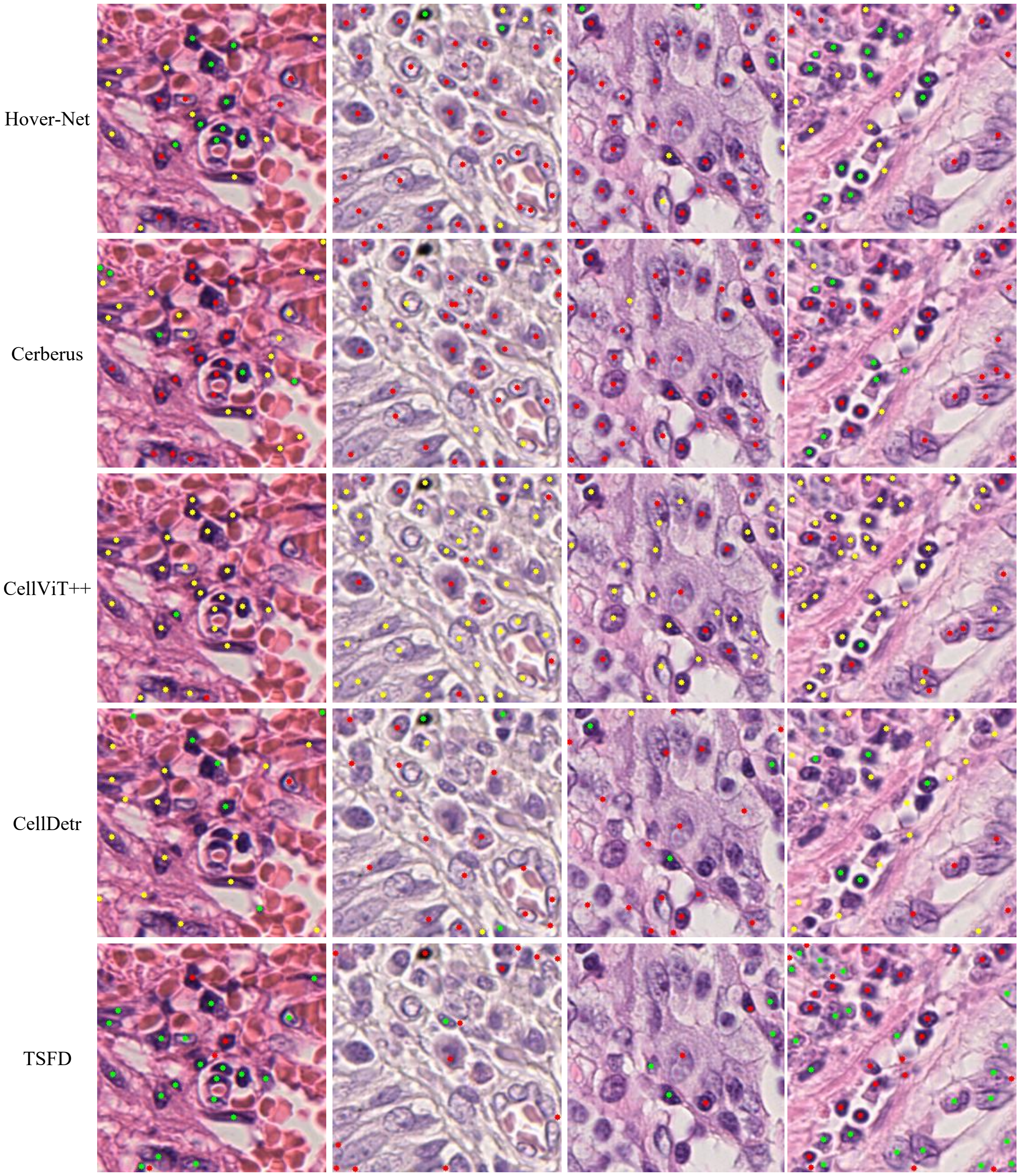
Hover-Net
Cerberus
CellViT++
CellDetr
TSFD

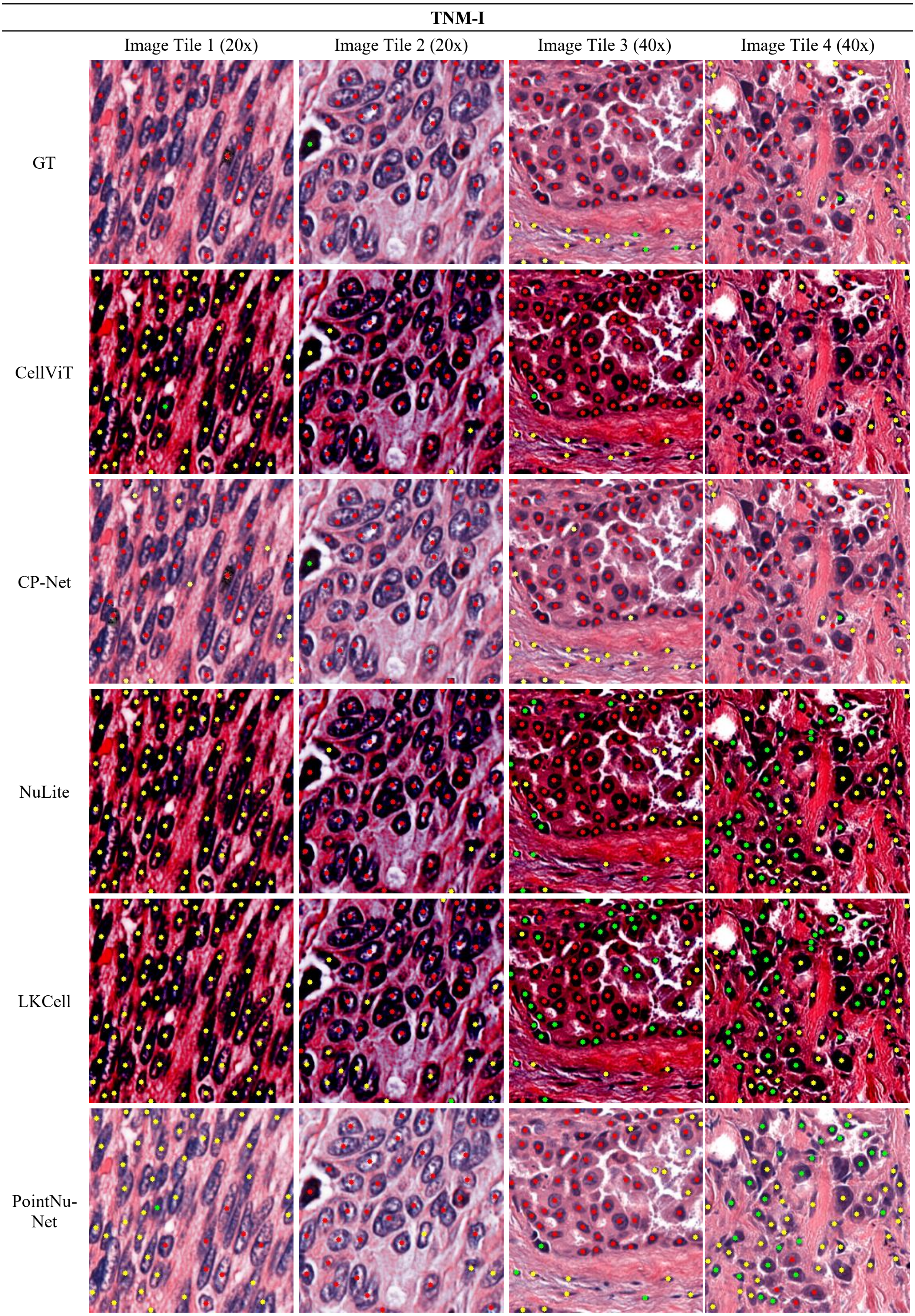

TNM-I
Image Tile 1 (20x)
Image Tile 2 (20x)
Image Tile 3 (40x)
Image Tile 4 (40x)
GT
CellViT
CP-Net
NuLite
LKCell
PointNu-Net

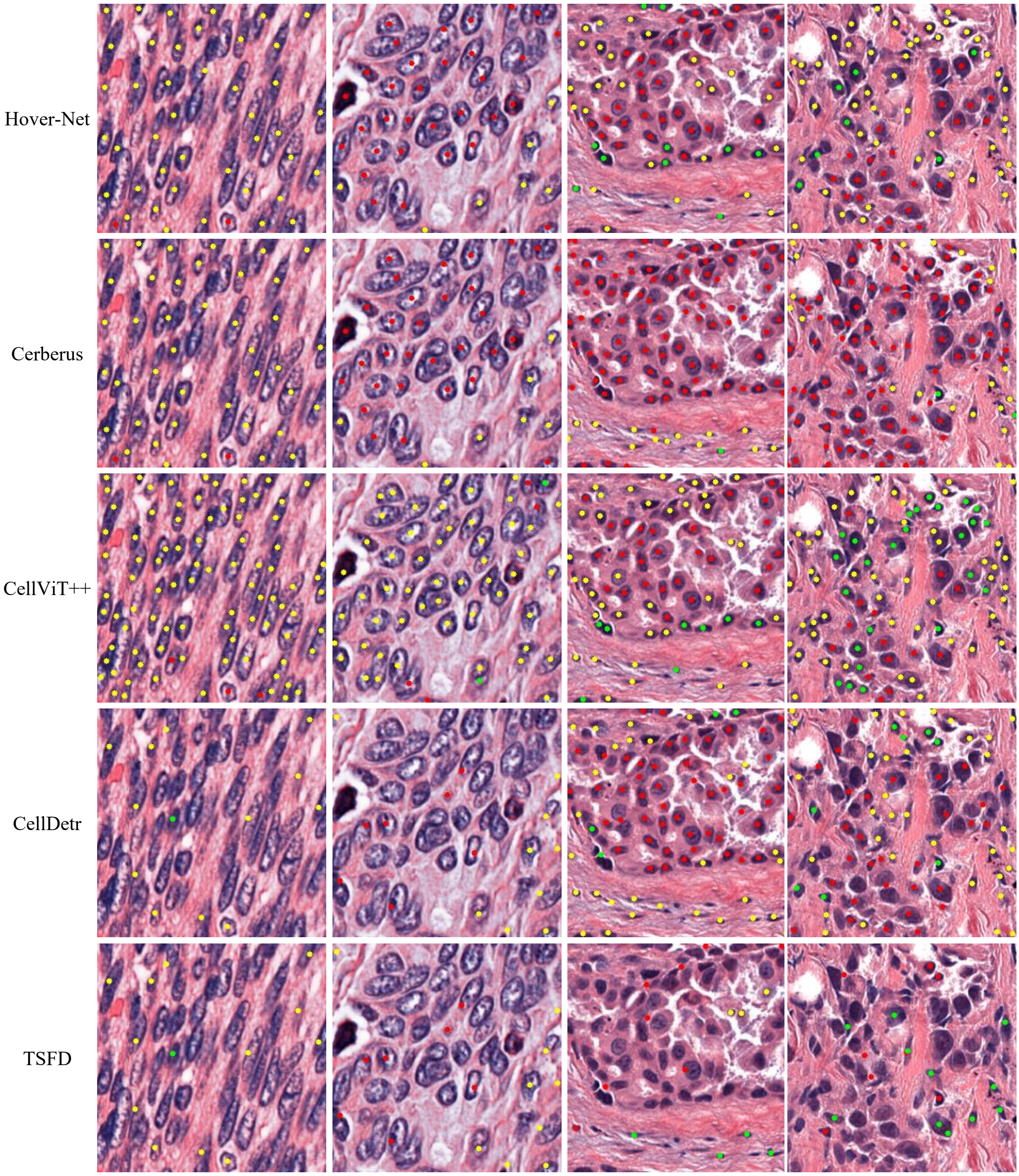

Hover-Net
Cerberus
CellViT++
CellDetr
TSFD

## References


1 S. Graham *et al.*, "Hover-Net: Simultaneous Segmentation and Classification of Nuclei in Multi-tissue Histology Images," *Med. Image Anal.*, vol. 58, no. 1, Dec. 2019, doi: 10.1016/j.media.2019.101563

2 S. Graham *et al.*, "Lizard: A Large-Scale Dataset for Colonic Nuclear Instance Segmentation and Classification," in *2021 IEEE/CVF International Conference on Computer Vision Workshops* , Montreal, BC, Canada: IEEE, Oct. 2021. doi: 10.1109/ICCVW54120.2021.00082

3 M. Amgad *et al.*, "NuCLS: A Scalable Crowdsourcing Approach and Dataset for Nucleus Classification and Segmentation in Breast Cancer," *Gigascience*, vol. 11, no. 1, May 2022, doi: 10.1093/gigascience/giac037

4 S. Liu, M. Amgad, D. More, M. A. Rathore, R. Salgado, and L. A. D. Cooper, "A Panoptic Segmentation Dataset and Deep-Learning Approach for Explainable Scoring of Tumor-Infiltrating Lymphocytes," *NPJ Breast Cancer*, vol. 10, no. 1, Jun. 2024, doi: 10.1038/s41523-024-00663-1

5 D. Komura *et al.*, "Restaining-Based Annotation for Cancer Histology Segmentation to Overcome Annotation-Related Limitations Among Pathologists," *Patterns*, vol. 4, no. 2, Feb. 2023, doi: 10.1016/j.patter.2023.100688

6 Hörst F, Rempe M, Becker H, Heine L, Keyl J, Kleesiek J. CellViT++: Energy-Efficient and Adaptive Cell Segmentation and Classification Using Foundation Models. Comput Methods Programs Biomed. 2026;277(1). doi: https://doi.org/10.1016/j.cmpb.2025.109206

7 G. Jevgenij, K. Navid Alemi, B. Ksenija, K. Ali, and R. Nasir, "PanNuke: An Open Pan-cancer Histology Dataset for Nuclei Instance Segmentation and Classification," *European Congress on Digital Pathology*, Apr. 2019, doi: https://doi.org/10.1007/978-3-030-23937-4_2

8 M. Rakaee *et al.*, "Machine Learning-Based Immune Phenotypes Correlate with STK11/KEAP1 Co-mutations and Prognosis in Resectable NSCLC: a Sub-study of the TNM-I Trial," *Annals of Oncology*, vol. 34, no. 7, Jul. 2023, doi: 10.1016/j.annonc.2023.04.005
